# Heme Oxygenase-1 at the Nexus of Endothelial Cell Fate Decision Under Oxidative Stress

**DOI:** 10.3389/fcell.2021.702974

**Published:** 2021-09-14

**Authors:** Sindhushree Raghunandan, Srinivasan Ramachandran, Eugene Ke, Yifei Miao, Ratnesh Lal, Zhen Bouman Chen, Shankar Subramaniam

**Affiliations:** ^1^Department of Bioengineering, University of California, San Diego, San Diego, CA, United States; ^2^Department of Diabetes Complications and Metabolism, City of Hope, Duarte, CA, United States; ^3^Department of Mechanical and Aerospace Engineering, University of California, San Diego, San Diego, CA, United States; ^4^Department of Computer Science and Engineering, University of California, San Diego, San Diego, CA, United States; ^5^Department of Cellular and Molecular Medicine, University of California, San Diego, San Diego, CA, United States

**Keywords:** oxidative stress, heme oxygease-1, HUVEC, ROS, hydrogen peroxide, HMOX1

## Abstract

Endothelial cells (ECs) form the inner lining of blood vessels and are central to sensing chemical perturbations that can lead to oxidative stress. The degree of stress is correlated with divergent phenotypes such as quiescence, cell death, or senescence. Each possible cell fate is relevant for a different aspect of endothelial function, and hence, the regulation of cell fate decisions is critically important in maintaining vascular health. This study examined the oxidative stress response (OSR) in human ECs at the boundary of cell survival and death through longitudinal measurements, including cellular, gene expression, and perturbation measurements. 0.5 mM hydrogen peroxide (HP) produced significant oxidative stress, placed the cell at this junction, and provided a model to study the effectors of cell fate. The use of systematic perturbations and high-throughput measurements provide insights into multiple regimes of the stress response. Using a systems approach, we decipher molecular mechanisms across these regimes. Significantly, our study shows that heme oxygenase-1 (HMOX1) acts as a gatekeeper of cell fate decisions. Specifically, HP treatment of HMOX1 knockdown cells reversed the gene expression of about 51% of 2,892 differentially expressed genes when treated with HP alone, affecting a variety of cellular processes, including anti-oxidant response, inflammation, DNA injury and repair, cell cycle and growth, mitochondrial stress, metabolic stress, and autophagy. Further analysis revealed that these switched genes were highly enriched in three spatial locations viz., cell surface, mitochondria, and nucleus. In particular, it revealed the novel roles of HMOX1 on cell surface receptors EGFR and IGFR, mitochondrial ETCs (MTND3, MTATP6), and epigenetic regulation through chromatin modifiers (KDM6A, RBBP5, and PPM1D) and long non-coding RNA (lncRNAs) in orchestrating the cell fate at the boundary of cell survival and death. These novel aspects suggest that HMOX1 can influence transcriptional and epigenetic modulations to orchestrate OSR affecting cell fate decisions.

## Introduction

Endothelial dysfunction is a hallmark of many cardiovascular diseases and can be triggered by oxidative stress (He and Zuo, 2015; Gimbrone and García-Cardeña, 2016). Endothelial cells (ECs) undergo oxidative stress in the presence of excess reactive oxygen species (ROS) such as hydrogen peroxide (HP), superoxide, and hydroxyl radicals. Uncompensated oxidative stress can cause significant damage to cellular macromolecules, disrupt cellular signaling, and lead to multi-faceted endothelial dysfunction, ranging from imbalanced redox signaling, impaired nitric oxide bioavailability, pro-inflammatory response to cell cycle disruption, and cell death. Persistent endothelial dysfunction and the extravasation of lipoproteins and monocytes into the sub-endothelial space is the first in a series of steps that leads to the formation of atherosclerotic plaque and, subsequently, more serious cardiovascular events (Winn and Harlan, 2005; Huynh et al., 2011; Zhou et al., 2013).

The change in the activity of cell signaling pathways, gene expression, and proteins that directly or indirectly moderate the response to damage by ROS is known as the oxidative stress response (OSR). In ECs, this involves the coordinated efforts by multiple signaling pathways (e.g., ATM/p53, NFκB, NRF2/KEAP1, and PI3K/Akt, etc.; Liang et al., 2017) that initiate changes in the cell’s structure and function culminating in the final phenotype (i.e., return to homeostasis, apoptosis, necrosis, or senescence). When oxidative stress overwhelms the protective responses, apoptosis may be induced, resulting in increased endothelial permeability (Winn and Harlan, 2005; Huynh et al., 2011; Zhou et al., 2013). Caspase-dependent apoptosis involves ROS-mediated (Elmore, 2007) activation of death receptors (FAS, TRAIL, etc.), mitochondrial cytochrome c release, or cytotoxicity resulting in the degradation of DNA by endonucleases (Chandra et al., 2000). Alternatively, the resistance to cell death can be established by anti-apoptotic factors such as protective members of the BCL2 family or the Inhibitors of Apoptosis (IAP) family of proteins (Portt et al., 2011). If the resistance to apoptosis is accompanied by cell cycle arrest, ECs can enter a quiescent or senescent state (Toussaint et al., 2002; Chen et al., 2008; Kuilman et al., 2010; Salminen et al., 2011; Muñoz-Espín and Serrano, 2014). EC fate decisions are critically important for determining vascular phenotypes. Quiescent cell cycle arrest is established during physiological conditions (Yu et al., 2001). Apoptosis facilitates vascular development and remodeling (Dimmeler and Zeiher, 2000; Mallat and Tedgui, 2000). EC proliferation is induced during angiogenesis and vascular repair (Aydogan et al., 2015). Heterogeneity in cell fate decisions among neighboring ECs can disrupt the monolayer and result in dramatic consequences for vascular health and function. While signaling pathways involved in the OSR and cellular pathways involved in cell fate decisions are well defined, these results are based on static models across various cell types and stress conditions providing limited temporal endothelial-specific insight.

Heme oxygenase-1 (HMOX1), an anti-oxidant enzyme activated by oxidative stress, is a central regulator of endothelial function and cell fate. The protective role of HMOX1 has primarily been attributed to its enzymatic activity in regulating concentrations of heme, CO, Fe^2+^, and biliverdin, which influence inflammation, redox status, expression of monocyte adhesion factors, and proliferation of ECs (Balla et al., 1991, 1993; Yachie et al., 1999; Brouard et al., 2000, 2002; Soares et al., 2002, 2004; Silva et al., 2006; Kim et al., 2007; Seldon et al., 2007; Gozzelino et al., 2010; Romanoski et al., 2011). Non-enzymatic activities of HMOX1 have also been shown to regulate the redox status and the expression of transcription factors associated with the OSR (Hori et al., 2002; Lin et al., 2007; Dulak and Jozkowicz, 2014). While the activation of HMOX1 has been observed as early as 2 h post-stress (Reichard et al., 2007; Chan et al., 2011) and in late-stage disease models (Deshane et al., 2005), its role in the transition from early to late endothelial dysfunction has not been explored.

This study provides a systems level analysis of the OSR in human umbilical vein endothelial cells (HUVECs) treated with HP. Using high throughput time-series gene expression measurements, we have identified three phases of the stress response consistent with known hallmarks of vascular disease. Next, we identified key players at the nexus of cell function and dysfunction, and HMOX1 as a central regulator of this transition. Further examination and perturbation of the transition from the early stress response to the prolonged stress response demonstrates the role of HMOX1 as the gatekeeper of cell fate decisions. Finally, we explore novel mechanisms by which HMOX1 coordinates stress signals affecting multiple cellular locations, culminating in the final decision between survival and apoptosis.

## Materials and Methods

### Cell Culture

Pooled HUVECs were purchased from ATCC (PCS-100-013, Lot#60279032) and Cell Applications (200p-05n). HUVECs were cultured in vascular cell basal media supplemented with an EC growth kit from ATCC (PCS-100,041). Cells used in this study were between passages 4–7.

### Hydrogen Peroxide Induction

The HP stock solution (30% ACS grade, 9.79 M, and Thermo Fisher #7722-84-1) was diluted to 100 mM in ice-cold HBBS (10.22 μl HP mixed with 989.88 μl HBSS), and it was further diluted to 5 mM (10× working concentration) in HBBS on ice. These dilutions were made just before the induction and used immediately. HP’s relative volume to growth media is 10%, to achieve the final concentration of 0.5 mM. 0.5 mM HP has been used in previous studies to examine a wide range of endothelial phenotypes such as growth and proliferation (Safaeian et al., 2017), anti-oxidant capacity (Jeon et al., 2015), cell death (Ni et al., 2013), and endothelial membrane blebbing (Gorp et al., 1999). Though the bulk concentration is 0.5 mM, the estimated HP concentration per cell is about 50 nM or 0.17 ng based on the number of cells at the induction time. About 5,000 cells/well were seeded at a time “0” in 100 μl of growth media. The media was replaced after 24 h and further incubated till 36 h before induction. The cell-doubling time for HUVEC’s was about 36 h. Therefore, about 10,000 cells/well (96 well plates) were estimated to be there at induction time.

### siRNA Knockdown Experiments

ATCC HUVECs were cultured to 50–60% confluence before being transfected with Lipofectamine RNAimax (Thermo Fisher, CAT# 13778150). Then, 48 h after the transfection, cells were treated with HP. 6 h after HP’s addition, cells were collected for analysis (phase-contrast imaging, qPCR, RNA-Seq, or Mito Stress test). Each knockdown experiment included Scrambled-Untreated [hereafter scrambled not-treated (scrNT)], Scrambled-Treated [hereafter scrambled treated (scrHP)], and Knockdown-Treated groups (ex: siHMOX1+HP). siRNA was obtained from Qiagen.

**Table:** 

Gene	Target sequence	Accession number	Catalog number
*HMOX1*	CAGGCAATGG CCTAAACTTCA	NM_002133	SI00278053
*ATF3*	CTGGGTGGTA CCCAGGCTTTA	NM_001030287	SI04168318
Negative control	–	–	1022076; SI03650325

### Sample Preparation for RNA-Seq

#### Time-Course Experiment

Human umbilical vein endothelial cells were cultured to near confluence and treated with 0.5 mM HP in duplicate. Samples were collected at 0 (control), 1, 2, 4, 6, 8, 10-, 12-, 14-, and 16-h time points. Total RNA was purified and concentrated using the Zymo Quick-RNA mini prep and RNA Clean & Concentrator kits (Zymo Research, #R1054 and #1015). Samples were sequenced on the Illumina HiSeq system (at the Salk Institute for Biological Studies, La Jolla, CA, United States).

#### Knockdown Experiment

Human umbilical vein endothelial cells were cultured and transfected with scrambled control or si-HMOX1 according to the Lipofectamine RNAiMAX protocol (13778075; Thermo Fisher, Waltham, MA, United States). After induction with HP, total RNA was isolated in duplicate using the Zymo Direct-zol RNA Miniprep Kit (R2050). Samples were submitted to the UC San Diego Genomics Core for stranded mRNA library preparation and sequencing on the Illumina HiSeq4000 system. scrNT was compared against scrHP to test the effect of HP treatment. Similarly, scrHP was used as a control to test the effect of the HMOX1 knockdown under HP treatment.

#### RNA-Sequencing Analysis

Both sequencing datasets include 50 base-pair single-end reads. The quality of the reads was verified using FastQC (Andrews, 2010). Next, Omicsoft Sequence Aligner 2 (OSA2; Hu et al., 2012) was used to align the reads to the Human.B37.3 reference genome build using the Ensembl R66 gene model resulting in gene counts. DESeq2 was used for differential expression analysis (Love et al., 2014). After identifying differentially expressed genes (DEGs), additional *p*-value (*p* < 0.01) and fold-change (|fold-change| > 1.3) cutoffs were implemented. Kyoto Encyclopedia of Genes and Genomes (KEGG) pathway (Kanehisa and Goto, 2000; Kanehisa et al., 2016, 2017), enrichment analysis was conducted using the DEGs list at each time point, assuming a hypergeometric distribution to calculate *p*-values. The enrichment of gene ontologies was also explored using DAVID (Huang et al., 2009). Transcription factor-target interactions were identified using the TRANSFAC database (Matys et al., 2006). Agglomerative hierarchical clustering was performed using the flashClust() function in R to cluster normalized gene counts (Langfelder and Horvath, 2012).

### Plate Assays

For the CCK8, Casp3/7, and GSH/GSSG plate assays, cells were seeded at ∼5,000 cells per well. Each assay included blank, negative, and positive controls (where applicable). Each plate assay had four technical replicates per dose. Fresh media was exchanged after 24 h, and cells were further incubated for 12 h before induction with different HP concentrations. For the CCK8, HUVECs were seeded in the black-walled, transparent bottom, 96-well plates (Greiner bio-one) in 100 μl of full growth media. For the Caspase Glo 3/7 and GSH/GSSG assays, HUVECs were seeded in the white-walled, opaque, 96-well plates (Greiner bio-one) in 100 μl of full growth media.

#### CCK-8 Assay

The CCK8 metabolic activity assay was used to measure dehydrogenase activity, a marker of cell metabolic activity. 10 μl of CCK-8 reagent (#CK04-11, Dojindo; Rockville, MD, United States) was added to each well at the end of 6, 12, 24, and 48 h of induction. Absorbance values were measured at 450 nm on the Tecan Safire M200 plate reader at 37°C. This experiment was repeated three times with four technical replicates per dose; the average blank value was subtracted from each treatment average and compared against a negative control (untreated cells) and expressed as percent toxicity. [*n* = 3 (P3–P5)].

#### Caspase-Glo 3/7 Assay

Caspase activity was measured with Caspase-Glo 3/7 reagent (G8090, Promega; Madison, WI, United States) 12 h after induction. Luminescence values were measured on the Tecan Safire M200 plate reader at RT and expressed as a percentage of the positive control (10 μM staurosporine). This experiment was repeated two times with four technical replicates for each dose [*n* = 2 (P5)].

#### GSH/GSSG-Glo Assay

GSH/GSSG-Glo kit (V6611, Promega; Madison, WI, United States) was used to assay the ratio of oxidized (GSSG) to reduced (GSH) glutathione. At the end of the induction time (12 h), 50 μl of Luciferin generating agent was added and incubated for 30 min at RT with intermittent shaking. Followed by 100 μl of luciferin detection reagent, luminescence was measured on the Tecan Safire M200 plate reader. This experiment included two independent experiments (*n* = 2) with three technical replicates per HP dose, along with vehicle control and no-cell control (blank). The average background measurement (from the blank) was subtracted from the average luminescence measurement for all other treatment groups and represented as a percentage of the untreated/vehicle control as per the manufacturer’s instructions.

#### Cell-Tox Green Assay

Cell toxicity was assayed with Cell-Tox reagent (G8742, Promega; Madison, WI, United States) at the end of 12 and 24 h of induction by microscopy. Inomycin (0.5 μM) was added to positive controls at the time of seeding. The assay reagent was added 30 min before the assay time and incubated at RT. Fluorescence was measured at 490 nm excitation and emission at 530 nm on the Olympus IX71 fluorescence microscope, three independent experiments (*n* = 3) with two technical replicates per HP dose.

#### Cell Cycle Analysis

Human umbilical vein endothelial cells were cultured and treated with 0 or 0.5 mM HP for 24 h. At the end of the treatment time, cell density was adjusted to 106 cells in 0.5 ml PBS and transferred to 4.5 ml of ice-cold ethanol overnight for fixing. Subsequently, the cell suspension was spun down (300 *g* at 4°C for 10 min), and ethanol was decanted. Next, the cell pellet was washed in 5 ml of PBS and incubated with 0⋅5 ml of staining solution (FxCycle PI-RNAse, LifeTechnologies) for 30 min in the dark before analyzing in the flow cytometer. Ten thousand events were collected for each sample, and their histograms were plotted for data visualization and analysis (*n* = 2). The percentage of cells in each phase with 0 and 0.5 mM treatment was compared using Student’s two-tailed *t*-test (*p* < 0.05 was considered significant).

#### Autophagy Assay

Cyto-ID (Enzo life sciences, United States) is an autophagy detection kit that selectively stains autophagic vacuoles without non-specific lysosome staining. HUVECs grown on glass-bottom dishes treated with 0 or 0.5 mM of HP for ≥24 h were stained with Cyto-ID as recommended by the manufacturer. After staining, cells were washed in wash buffer supplemented with 5% FBS and imaged in wash buffer. Autophagic vacuoles were imaged with excitation at 463 nm and emission at 534 nm (*n* = 2, each with two technical replicates per treatment condition).

#### Mitochondrial Network Integrity

HUVECs plated on glass-bottom Petri dishes (MatTek Corp.) at 5,000 cells/cm^2^ were allowed to reach 70% confluence before induction with 0 and 0.5, 0.6, and 0.7 mM HP for 12 and 24 h. Cells were stained with MitoTracker Deep Red FM dye (10 nM) mixed with Hoechst 33342 (0.75 μg/ml; LifeTechnologies, United States) in warm fresh media for 45 min at 37°C. Cells were washed in warm media and imaged in warm HBSS in an environment-controlled chamber on IX71 microscope. 3–5 random fields were chosen for 100x imaging. Acquisition parameters were optimized for control cells, and the same parameters were used for acquiring all treatment groups. 3D stacks (*X*, *Y*, and *Z*) for each channel (Blue and Red) were obtained and combined in Metamorph Imaging software. Images were analyzed in ImageJ software. To visualize the mitochondrial network, their distribution, and size, a high pass filter (edge detection) was applied, followed by the maximum intensity projection to determine the total intensity of the mitochondrial stain (*n* = 2 with two technical replicates per treatment condition).

#### Mitochondrial Membrane Potential Assay

Cells grown on glass-bottom dishes were treated with different HP concentrations for 12 h, subsequently incubated with 5 μM JC-1 (Biotium, United States) diluted in warm fresh media for 30 min at 37°C. After this, cells were gently washed with two volumes of fresh media two times- each for 3 min and imaged immediately on IX71 equipped with an environmental chamber. For JC-1 monomers and aggregates, excitation and emission wavelengths of 485/535 and 560/595 nm, respectively, were used. Mitochondrial membrane depolarization is indicated by green fluorescent monomers (unhealthy/apoptotic cells), and red fluorescent aggregates indicate healthy mitochondria. Ratios of monomers/aggregates (green/red ratio) indicate the overall health of mitochondria in the treatment group. Images were analyzed in ImageJ using a macro that automatically segments and quantifies green and red fluorescence. (*n* = 2 with two technical replicates per HP dose).

#### Quantitative PCR

RNA was isolated from cells using Trizol (Thermo Fisher, 15596018). Total RNA was reverse transcribed using the Clontech Prime Script RT Master Mix (RR036B) followed by qPCR with SYBR Green (Bio-Rad); qPCR was run Bio-Rad CFX-96 realtime system. Beta-Actin and GAPDH were used as the housekeeping genes (HKGs) when calculating the delta Ct values.

**Table:** 

Gene	Forward primer	Reverse primer
MT-ND3	GCGGCTTCGACCCTATATCC	AGGGCTCATGGTAGGGGTAA
MT-ATP6	TCCCTCTACACTTATCATCTTCAC	GACAGCGATTTCTAGGATAGTC
IGF1R	CCTGCACAACTCCATCTTCGTG	CGGTGATGTTGTAGGTGTCTGC
EGFR	TATGTCCTCATTGCCCTCAACA	CTGATGATCTGCAGGTTTTCCA
RBBP5	GTGGACCCTATTGCTGCCTTCT	CCATCAAGGAGGTTTGGACTGC
KDM6A	AGCGCAAAGGAGCCGTGGAAAA	GTCGTTCACCATTAGGACCTGC
PPM1D	GTGGTCATCATTCGGGGCAT	CATCCTTCGGGTCATCCTGAA
HMOX1	AAGACTGCGTTCCTGCTCAAC	AAAGCCCTACAGCAACTGTCG
ATF3	CCTCTGCGCTGGAATCAGTC	TTCTTTCTCGTCGCCTCTTTT
B-ACTIN	GCACCACACCTTCTACAATG	ATCACGATGCCAGTGGTAC
GAPDH	CTCCTCACAGTTGCCATGTA	GTTGAGCACAGGGTACTTTATTG

### Statistical Analysis and Plotting of qPCR Results

Delta-Delta-Ct calculations were carried out in Microsoft Excel: First, the average Ct values of each gene of interest (GoI) and the HKGs in each experimental condition were calculated. Then, Delta Ct (ΔCt) was determined by subtracting the average HKG Ct from the average GoI Ct values. Next, Delta-delta-Ct was calculated by subtracting the ΔCt values of scrNT sample from scrHP. Similarly, ΔCt values of scrHP were subtracted from siHMOX1+HP (siRNA knockdown of HMOX1 cells treated with HP). Finally, statistical comparisons and plotting were carried out in R (v. 4.0.5) using the “ggstatsplot” package (v. 0.8.0; Patil, 2021). This package determines the number of observations, validates their normality and variance, and carries out appropriate statistical tests and reports the type of test used, test statistic, *p*-value, bias-corrected effect size $\hat{g}$, 95% CI and number of observations. It also reports the likelihood ratio of the null hypothesis (H_0_) over the alternate hypothesis (H_1_) as log_e_ (Bayes Factor_01_). In addition, it determines the appropriate geometry for graphing the underlying data combined with statistical analysis in a single plot. Since the plots are relatively large, we are presenting them in the Supplementary Material.

#### Quantifying Mitochondrial Respiration (Seahorse Mito Stress Test XFe24)

Mitochondrial function was assessed using the Mito Stress Test (XFe24, Agilent). After 48 h of transfection, cells were trypsinized, resuspended, and plated into the XFe 24-well plate at a density of 30,000 cells/well. The cells were allowed to grow overnight before being treated with HP. 5 h after treatment, the cell culture media was removed and replaced with the Agilent base media (102353–100, Agilent; Santa Clara, CA, United States) supplemented with D-glucose (5.5 mM) and L-glutamine (0.68 mM) before being incubated in a non-CO_2_ incubator for 1 h. Oligomycin, FCCP, and Antimycin/Rotenone were loaded into ports A, B, and C, respectively, of the cartridge at the following concentrations: Oligomycin (1 μM), FCCP (2 μM), and Ant/Rot (0.5 μM). All oxygen consumption measurements were normalized by cell count or protein concentration (BCA Protein Quantification). This experiment was repeated three times (*n* = 3, P5–7). First, the non-mitochondrial oxygen consumption (average Rot/AA) is subtracted from the basal, oligomycin and FCCP average measurements to generate their corresponding adjusted parameters for each treatment group (scrNT, scrHP, and siHMOX1+HP). Then, mitochondrial function parameters were calculated as follows: ATP production = Adj. Basal – Adj. Oligo; Proton Leak = Adj. Oligo; Spare Capacity = Adj. FCCP – Adj. Basal. Statistical comparisons were made across scrNT vs. scrHP; and scrHP vs. siHMOX1+HP for various mitochondrial respiratory parameters, including spare capacity, ATP-linked mitochondrial respiration and proton leak. Again, a threshold of *p* < 0.05 was used to identify significant changes.

#### Quantitative Determination of Hydrogen Peroxide

The assay was carried out per the manufacturer’s (Enzo Catalog #: ADI-907-015) recommended protocol. Briefly, different endothelial culture media compositions [Basal media, Basal media + Growth factors, Basal media + Serum, Basal media + Growth factors + Serum (complete growth media), phosphate-buffered saline] were prepared. Fifty microliters of each of these samples were mixed with 50 μl of 200 μM HP provided in the kit and 100 μl of the color reagent. After mixing gently, sample absorbance at 550 nm was recorded at different time points (0, 10, 20, 30, 40, 50, and 60 min), and the average net absorbance is plotted after subtracting the blank from the measured values. A standard curve was generated with the standard solution provided in the kit before measuring the sample absorbance.

## Results

### Dose and Time-Dependent Response to Oxidative Stress in HUVECs

To study the cellular response to oxidative stress, specifically at the boundary of cell survival and death, we explored the mechanisms that follow HP-induced stress in HUVECs. We examined the dose-response of ECs using functional readouts such as metabolic activity, anti-oxidant capacity, caspase 3/7 activation, and cell toxicity to characterize different aspects of cellular response.

**FIGURE 1:**
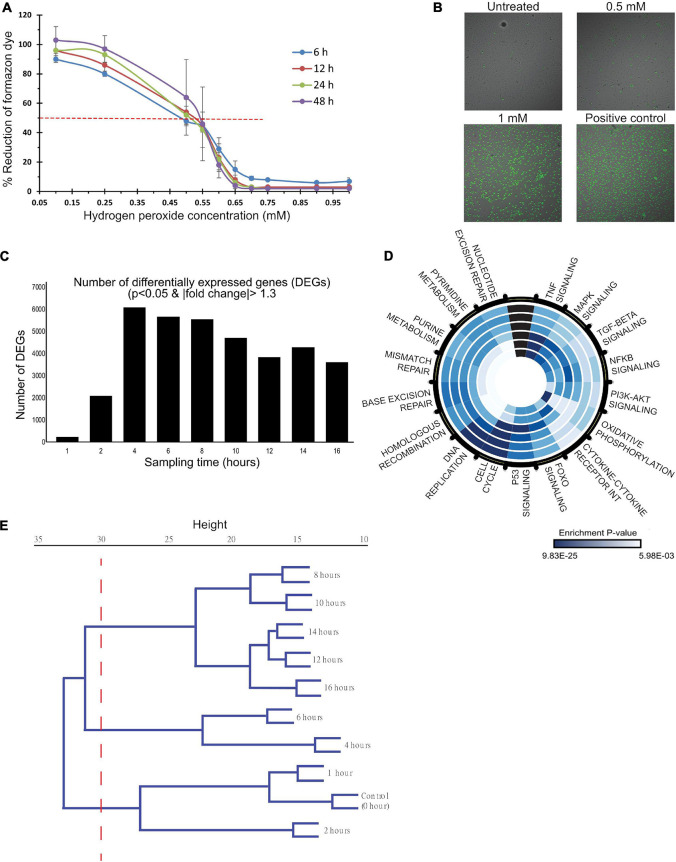
A dose- and time-dependent analysis of HP induced oxidative stress in HUVECs. **(A,B)** HUVECs were treated with 0–1 mM HP, and functional measurements were made to characterize the dose-dependent response to oxidative stress. **(C–E)** mRNA samples, collected at 0, 1, 2, 4, 6, 8, 10, 12, 14, and 16 h post-treatment (0.5 mM HP), were sequenced to characterize the time-dependent response to oxidative stress. **(A)** Dehydrogenase activity was measured as a proxy for cell metabolic capacity. Blank adjusted measurements are reported as a percentage of the negative control (untreated cells) at 6, 12, 24, and 48 h post-treatment. Three biological experiments (*n* = 3), each had four technical replicates. **(B)** Cyto-Tox Green assay: the amount of green staining is proportional to cytotoxicity. Scale bar: 20 μm. Three biological experiments (*n* = 3), each had two technical replicates. **(C)** The number of differentially expressed genes at each time point post-HP treatment to control (0 h); DE genes were identified by a *p*-value cutoff of 0.01 and a | fold-change| cutoff of 1.3. **(D)** Significantly enriched KEGG pathways. Each ring of the circle represents one time-point, where the inner-most ring represents 1 h, and the outer-most ring represents 16 h. Dark blue indicates highly significant *p*-values, whereas light blue represents less significant *p*-values. **(E)** Hierarchical clustering dendrogram of normalized gene counts from each time point.

To determine the appropriate dose and time window to characterize the OSR, dose-response and time-series measurements were carried out using CCK-8 assay that measures cell metabolic capacity through intracellular dehydrogenase activity. Cell metabolic activity decreased across all time points with increasing HP dose and is reduced to 50% between 0.5 and 0.55 mM (Figure 1A). Doses beyond 0.6 mM drastically reduced their metabolic capacity. To verify that 0.5 mM establishes significant oxidative stress, the ratio of reduced to oxidized glutathione (GSH/GSSG), a specific measure of cellular anti-oxidant capacity, was measured at 12 h. It showed a reduced anti-oxidant capacity by 50% (Supplementary Figure 1A). Similarly, caspase3/7 assays showed a positive trend of a dose-dependent increase in activity up to 0.5 mM (Supplementary Figure 1B) with no caspase activity beyond 0.65 mM HP. Suggesting caspase-dependent cell death mechanisms at low doses and caspase-independent mechanisms at higher doses. In addition to damaging proteins and DNA, ROS also disrupts the integrity of lipid membranes. Cell toxicity was measured to ensure that the induction dose does not result in drastic cell death as it affects the quality of genomic material for gene expression studies. Cell-Tox green assay showed increasing toxicity (number of green cells) with dose. 24 h after induction, about 10% of cell death was observed (Figure 1B). Overall, these assays established that 0.5 mM HP induces significant oxidative stress and places the cell at the transition of cell function and dysfunction.

To characterize the mechanisms at this transition, total RNA was collected at 0, 1, 2, 4, 6, 8, 10-, 12-, 14-, and 16-h post-treatment with 0.5 mM HP for RNA-sequencing. The number of DEGs at each time point is shown in Figure 1C. There was a dramatic increase in the number of DEGs to ∼6,000 between 1 and 4 h, and it gradually decreased to ∼3,500 between 4 and 16 h. Summary of data quality and the exact number of reads sequenced per sample, and the uniquely mapped alignment percentage are summarized in Supplementary Figure 2.

Kyoto Encyclopedia of Genes and Genomes pathway enrichment was carried out to identify the significant processes across the time course (Figure 1D). Early time-points (<4 h) were enriched for TNFα, MAPK, TGFβ, NFκB, PI3K/Akt, and FOXO signaling along with pathways related to cytokine-cytokine receptor interaction and oxidative phosphorylation. At 4 h, p53 signaling was maximally enriched. From 4 to 16 h, cell cycle pathways, DNA replication, and DNA repair were enriched.

Hierarchical clustering of the normalized gene counts identified distinct phases of the OSR (Figure 1E). At a tree height cutoff of ∼30, three distinct clusters emerged: 0–2 h defined the “early” cluster, 4–6 h formed the “mid/transitional” cluster, and 8–16 h formed the “late” cluster. Additional functional analyses showed a trend of decreased and increased cell populations in the S phase and G2/M phases, respectively (Supplementary Figures 3A,B). Also, there was an increase in autophagic vacuoles to HP treatment (Supplementary Figure 3C). Mitochondrial analysis showed a trend of increase in membrane depolarization, decrease, and disintegration of its extended network into small fragments upon HP treatment (Supplementary Figures 3D–F).

Taken together, the dose- and time-series response to oxidative stress (Figure 1) suggest a nexus between cell function and dysfunction, and it is characterized by three distinct phases (early, mid, and late). The activation of classical OSR in the early phase is later accompanied by cell cycle regulation, DNA replication, and DNA repair pathways.

### A Temporal Model of the Oxidative Stress Response in Endothelial Cells

**FIGURE 2:**
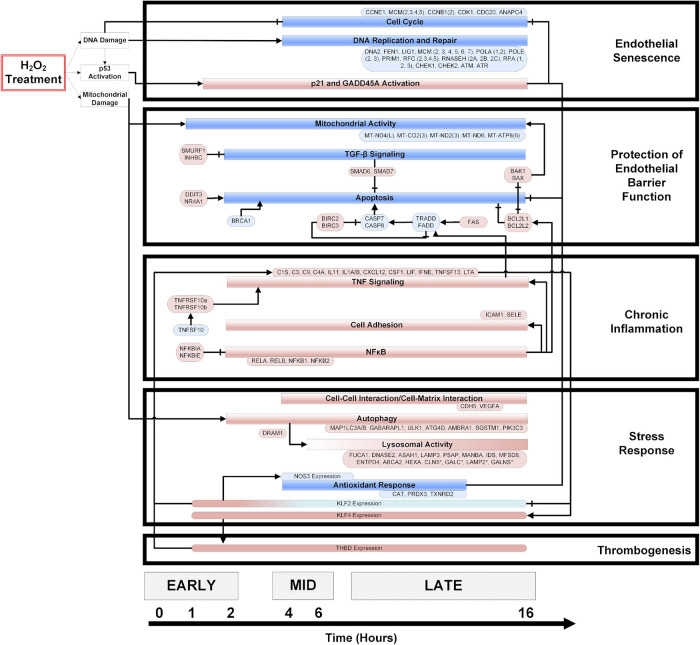
A temporal model of HP induced oxidative stress in HUVECs. HUVECs treated with 0.5 mM HP in duplicate, and mRNA samples (from 0 to 16 h) were sequenced. Significantly regulated genes and cellular processes (*p* < 0.05) were used to construct a temporal model depicting oxidative stress response time-course. The boxes colored in blue and red represent down- and up-regulated genes or processes, respectively. The axis at the bottom of the figure indicates the time at which various processes become significantly regulated. An arrowhead depicts positive regulation, and a flat line indicates negative regulation. Vascular phenotypes associated with each group of genes/processes (i.e., endothelial senescence, protection of endothelial barrier function, chronic inflammation, stress response, and thrombogenesis) are indicated to the right of the temporal model. The log_2_ (fold-change) values for the genes specified in this model are summarized in the Supplementary Figure 3.

Significantly enriched genes and pathways (*p* < 0.05) were identified from the time-series gene expression measurements and used to construct a temporal model of the OSR (Figure 2). The direction of the pathway and gene regulation is specified: blue indicates down-regulation, and red indicates up-regulation (the exact fold-change and *p*-values are summarized in Supplementary Figure 4). In the first hour, we observed gene expression changes associated with mitochondrial damage and p53 activation. Mitochondrial damage was characterized by the consistent downregulation of key mitochondrial transcripts associated with the electron transport chain (ETC) subunits and ATP synthase from 1 to 16 h. At 2 h, potent cell cycle inhibitors [CDKN1A (p21) and GADD45A] were up-regulated and remained up-regulated until 16 h. Further, a strong down-regulation of cell cycle genes (cyclins, CDKs, etc.) was observed from 4 to 16 h. Interestingly, DNA replication and repair genes were also strongly down-regulated from 4 to 16 h.

We observed a balance between pro-and anti-apoptotic gene programs. The resistance to apoptosis is maintained by strong upregulation of the IAP family members BIRC2 and BIRC3. Anti-apoptotic BCL family members BCL2L1 and BCL2L2 were also strongly up-regulated, and pro-apoptotic factors such as BRCA1, CASP8, and Caspase inducing TRADD and FADD were down-regulated across time. Finally, the upregulation of TGFβ family members SMAD6 and SMAD7 also supported the resistance to apoptosis. On the other hand, pro-apoptotic factors such as FAS, CHOP, NR4A1, BAK1, and BAX were strongly up-regulated across the time course (Figure 2).

Pro-inflammatory processes such as NFκB signaling and expression of cytokines and chemokines were up-regulated from 2 to 16 h. Interestingly, inhibitors of NFκB (NFKBIA, NFKBIB) are also up-regulated across the time course. In addition, ICAM1, known to recruit and facilitate pro-inflammatory monocyte extravasation, was up-regulated from 2 to 16 h.

NFE2L2, a critical transcription factor involved in the OSR, is up-regulated from 2 to 8 h and again at 12–14 h. Key autophagy proteins such as LC3A and PIK3C3 were up-regulated from 2 to 16 h, while other autophagic proteins such as ATG4C were down-regulated from 4 to 10 h. Lysosomal activity increased over time, with a majority of lysosome hydrolases being up-regulated at 16 h.

### HMOX1: A Key Regulator

**FIGURE 3:**
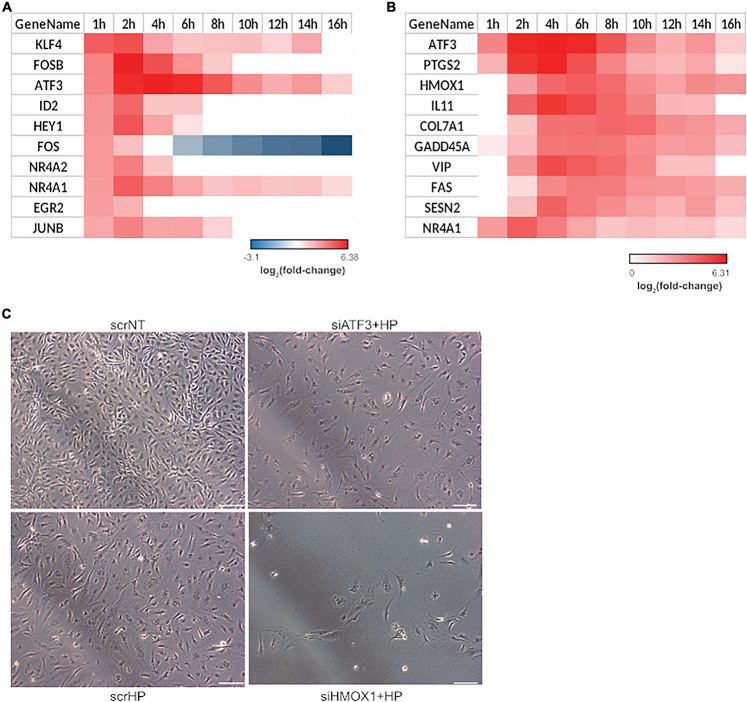
HMOX1 is a key regulator of cell fate in HUVECs upon HP treatment. Significant transcription factor-target interactions were identified to determine key gatekeepers of cell fate and function. **(A)** Top 10 up-regulated transcription factors at 1-h post HP treatment. **(B)** The top 10 up-regulated gene targets of JUN family transcription factors. All JUN targets differentially expressed at least one-time points were identified and ranked by average fold-change across all time points. **(C)** Two genes were selected from **(B)** for a siRNA screen experiment: ATF3 and HMOX1. After 48 h of transfection and 6 h of HP treatment, HUVECs were imaged using phase-contrast microscopy (the images shown are the representative images taken from 3 repeats of the experiment; scale bars indicate 100 μm).

Since the phenotype switch occurred from 4 to 6 h as indicated by the hierarchical clustering and functional analysis, transcription factors induced in the first hour that may facilitate the switch from 4 to 6 h were identified. Of the top ten transcription factors that were up-regulated, four were JUN family transcription factors: FOSB, ATF3, FOS, and JUNB (Figure 3A). To explore the mechanistic consequence of JUN activation, the top ten up-regulated JUN family targets were identified using the TRANSFAC database (Matys et al., 2006; Figure 3B). From this list, two gene candidates (i.e., HMOX1 and ATF3) were selected based on the following criteria: (1) their involvement with the regulation of oxidative stress-related processes (Han et al., 2008; Yoshida et al., 2008; Gozzelino et al., 2010; Tanaka et al., 2011) and (2) association with endothelial apoptosis (Kawauchi et al., 2002; Soares et al., 2002). Additionally, it is important to note that the role of HMOX1 and ATF3 in the transition from acute to chronic stress response has not been explored. The knockdown experiments included three treatment groups: (1) scrNT, (2) scrHP, and (3) knockdown-treated. We observed no significant differences in cell count between the scrambled siRNA and siHMOX1 transfected cells prior to the addition of HP (Figure 3C). Statistical analysis of HMOX1 and ATF3 knockdown in HP treatment is shown in Supplementary Figure 5. Both HMOX1 and ATF3 showed significant *p*-values (0.002 and 0.01), very strong bias adjusted effect size $\hat{g}$ (4.84 and 4.39) and strong evidence for the alternate hypothesis (Bayes factor), log_e_(BF_01_; −3.64 and −3.31), respectively. The comparison of scrHP vs. scrNT displays the effect of the HP treatment; we observed a decrease in cell count and a change in cell morphology. Comparing the scrHP with each knockdown +HP group revealed that the greatest change in cell count and morphology occurred with HMOX1 knockdown.

### HMOX1: A Global Regulator of Cell Function

**FIGURE 4:**
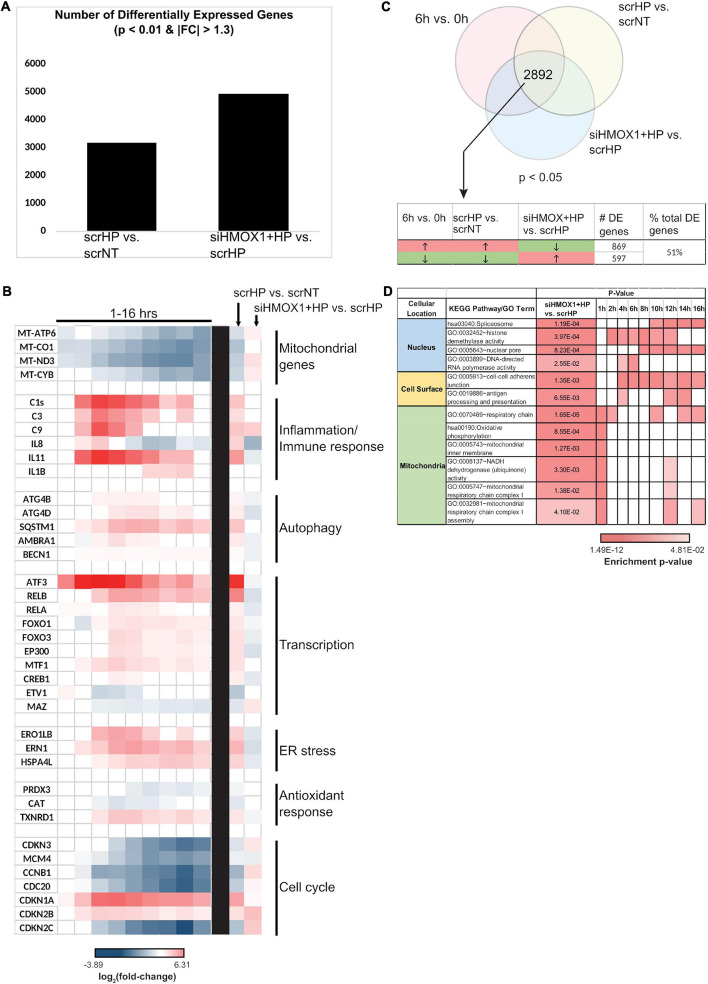
HMOX1 is a global regulator of cell function in HUVECs upon HP treatment. RNA collected from scrNT, scrHP, and siHMOX1+HP samples (*n* = 2 per group) was submitted for RNA-seq. **(A)** The number of differentially expressed genes between scrHP vs. scrNT (i.e., indicating the effect of the HP treatment) and between siHMOX1+HP vs. scrHP (i.e., indicating the effect of the HMOX1 knockdown in response to HP treatment). **(B)** Log_2_ (fold-change) of genes associated with mitochondrial function, inflammatory/immune response, autophagy, transcription, ER stress, anti-oxidant response, and cell cycle in the time-course data (to the left of the black bar) and the knockdown experiment (to the right of the black bar). **(C)** The Venn diagram indicates the number of overlapping differentially expressed genes between the time-course experiment and the knock-down experiment (i.e., the 6 vs. 0 h time-course data and the treatment groups; *p* < 0.05). The table indicates the number and percentage of overlapping genes that switch the direction of expression upon HP treatment in HMOX1 deficient HUVECs. **(D)** KEGG and GO enrichment analysis of the overlapping genes that switch their direction of regulation upon HP treatment in HMOX1 deficient HUVECs. Enrichment of pathways in the time-course data is also shown. The cell’s color indicates the significance of the *p*-value (the lighter the shade, the less significant, and the darker the shade, the more significant the *p*-value). The enriched KEGG pathways and GO terms were grouped into three spatial categories, as specified in the table’s first column.

The global role of HMOX1 in orchestrating the OSR in the transitional regime between early and late stress was examined by transcriptomic analysis. Measurement of HMOX1 expression upon HP treatment by qPCR between scrNT and scrHP showed ∼fourfold induction, while comparison between scrHP and siHMOX1+HP showed knockdown efficiency (∼80%). After alignment with OSA2, gene counts were used for differential expression analysis (DEGs are summarized in Figure 4A).

Key processes affected by HP treatment were examined in the knockdown context (Figure 4B). Many processes that were up-regulated by HP treatment alone were down-regulated in HMOX1 deficient cells and vice versa. For example, mitochondrial transcripts MT-ATP6, MT-ND3, and MT-CYB were all strongly down-regulated upon HP treatment alone. However, after the knockdown, these transcripts were up-regulated. Similarly, genes associated with inflammation, autophagy, transcription, ER stress, anti-oxidant response, and cell cycle switch their direction of expression in response to HP when HMOX1 expression was decreased. Among the 2892 DEGs (*p* < 0.05), about 51% of them switched their direction of expression upon HMOX1 knockdown in the HP treatment group (Figure 4C). GO and KEGG enrichment of these switched genes revealed enrichment in three spatial regions: nucleus, cell surface, and mitochondria (Figure 4D). These data suggest the global impact of HMOX1 on regulating the functional response to oxidative stress.

### Novel Mechanisms of HMOX1 in Regulating the OSR

**FIGURE 5:**
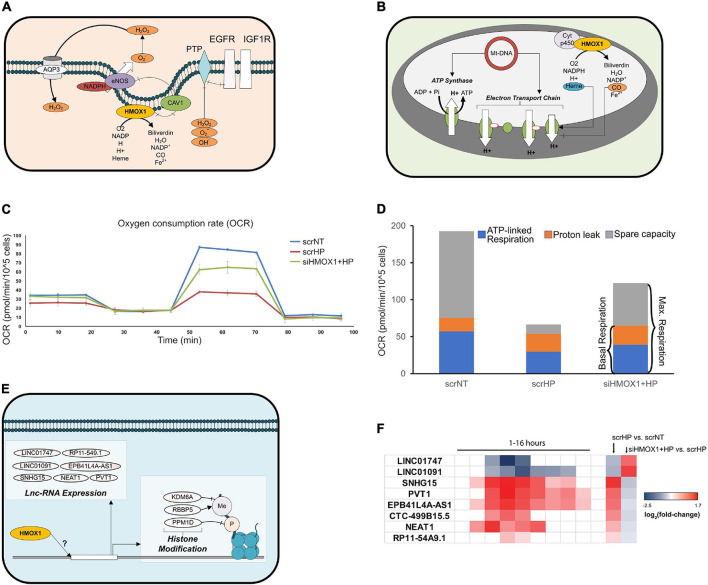
HMOX1 deficiency perturbs the OSR at the cell surface, nucleus, and mitochondria. Differentially expressed genes and pathways in each cellular location were examined to identify novel targets of HMOX1 regulation. **(A)** Network diagram depicting HMOX1 activity at the cell surface. **(B)** Network diagram depicting HMOX1 activity in the mitochondria. **(C)** The Mito Stress Test (Seahorse XFe24) was used to quantify functional changes in mitochondrial function. The line-graph shows a representative oxygen consumption rate (pmol/min/cell) for one of the three biological repeats of this experiment (*n* = 3); 1 μM Oligomycin was added at ∼20 min, followed by 2 μM FCCP at ∼50 min and finally 0.5 μM Rot/AA at ∼80 min. The background-subtracted mean OCR + SEM across two to four technical replicates for scrNT (blue line), scrHP (red line), and siHMOX1+HP (green line) is shown. **(D)** The stacked bar graph shows the proton leak, ATP production and spare capacity across all three groups. **(E)** Network diagram indicating lnc-RNAs and histone modification genes affected by HMOX1 expression. **(F)** Log_2_ (fold-change) of lnc-RNAs from the time-course experiment and the knockdown experiment, indicating the switch in expression upon HMOX1 deficiency. *p* < 0.05 are shaded; *p* > 0.05 are blank. Log_2_ (fold-change) and *p*-values for genes described in panels **(A,B,E)** are summarized in Supplementary Figure 6.

Upon further analysis of HMOX1 knockdown (KD) location-specific effects, key mitochondrial transcripts, cell surface receptors, and nuclear regulatory genes switched their direction of expression upon HP treatment. At the cell surface, EGFR and IGF1R were up-regulated with HP treatment in HMOX1 competent and down-regulated in HMOX1 KD cells (Figure 5A and Supplementary Figure 6). Mitochondrial transcripts that encode the ETC subunits were down- and up-regulated upon HP treatment in HMOX1 competent and KD cells, respectively. This is further validated by qPCR (Supplementary Figure 7). To gauge this transcriptional switch’s functional consequence, we performed the Seahorse Mito Stress Test to quantify mitochondrial respiration. Consistent with the transcriptional switch, measurement of the oxygen consumption rate in the scrNT, scrHP, and siHMOX1+HP groups indicated a decrease in spare capacity upon HP treatment, reversed in the HMOX1 KD group (Figures 5C,D and Supplementary Figure 8). The decrease in ATP-linked respiration (scrNT vs. scrHP, *p* = 0.03) is ameliorated in the HMOX1 KD group but not statistically significant.

Finally, we showed that key histone modification genes (KDM6A, RBBP5, and PPM1D) were up-and down-regulated upon HP treatment in HMOX1 competent and deficient cells, respectively (Figure 5E and Supplementary Figures 6, 9). Additionally, we have identified lncRNAs that switched their direction of expression in response to HP treatment in the HMOX1 KD group (Figure 5F and Supplementary Figure 6). Taken together, these results highlight novel organelle-specific HMOX1-mediated mechanisms of cell protection in response to oxidative stress.

## Discussion

Endothelial dysfunction and cell death are central to the progression of vascular diseases (Madamanchi et al., 2005; Libby et al., 2011; Gimbrone and García-Cardeña, 2016). Under chronic stress, gaps in the endothelium allow uninhibited extravasation of immune cells and lipoproteins, contributing to the persistent pro-inflammatory state that supports plaque formation (Galbraith et al., 1998). Endothelial dysfunction and cell death can be triggered by many mechanical (Chien, 2008; Chiu and Chien, 2011; Ajami et al., 2017) and chemical cues (Hadi et al., 2005; Deanfield et al., 2007). ECs are primed to sense and respond to subtle chemical perturbations such as oxidative stress as the first line of defense to the circulating toxins in the blood (Deanfield et al., 2007; Pennathur and Heinecke, 2007). In this study, we have characterized the response to oxidative stress in ECs using a systems biology approach. In doing so, we have identified gatekeepers at the nexus of cell fate decisions.

### Dose-Dependent OSR in ECs

One of the critical features of vascular disease is eNOS uncoupling, which results from the oxidation of nitric oxide; this process is dependent on the amount of ROS available (i.e., the degree of oxidative stress; Elahi et al., 2009). We have shown the dose dependence in metabolic activity and cell toxicity (Figures 1A,B; Clément et al., 1998; Gorp et al., 1999; Csordas et al., 2006; Gong et al., 2010; Messner et al., 2012; Wen et al., 2013; Xu et al., 2013; Zhou et al., 2013; Nadeev et al., 2015). At 0.5 mM HP, the metabolic and anti-oxidant capacities are reduced by 50%, which induces significant oxidative stress. Also, HP is unstable in growth media. About 50% of added HP is cleared in less than 20 min, and it is undetectable around 60 min (Supplementary Figure 10), even without cells. Therefore, a single bolus of 0.5 mM HP induction perturbs the cell, places it at the junction of cell survival and death, and serves as a model to study early and late phase OSR.

### Time-Dependent OSR in ECs and Hallmarks of Vascular Disease

We examined the longitudinal OSR to characterize transcriptional programs that facilitate the transition from EC function to dysfunction. In addition, our dose-response analysis revealed the ECs’ functional tipping point, allowing us to focus on factors that initiate cell dysfunction. We hypothesized that the temporal OSR would be best reflected by the transcriptional state of the cell and hence measured the longitudinal gene expression changes through next generation RNA sequencing. Analysis of the expression profile data allowed us to distinguish three distinct phases of the stress response and provide a systems perspective of OSR in ECs (Figure 2).

We observed both pro-and anti-apoptotic mechanisms as part of the global response to oxidative stress. This ambiguity is likely due to the heterogeneous cell populations present in the culture and/or the concurrent activation of pro-and anti-apoptotic processes in individual cells, highlighting the complexity of stress response. Our model finds that the down-regulation of cell cycle and DNA replication is correlated with the induction of p21 and GADD45A from 4 to 16 h (Figure 2 and Supplementary Figure 4); this is in line with previous findings (Zhan, 2005; Gao et al., 2009; Moskalev et al., 2012; Niehrs and Schäfer, 2012; Tamura et al., 2012). Further, in addition to cell cycle arrest, our data reveal that hallmarks of endothelial senescence, such as up-regulation of pro-inflammatory cytokines, monocyte adhesion factors, and autophagy along with down-regulation of NOS3, in a sustained manner throughout the time course (Lum and Roebuck, 2001; Tian and Li, 2014). Therefore, at the nexus of cell function and dysfunction, decreased replicative potential and activation of senescence supports key pillars of endothelial dysfunction such as decreased NO, increased cytokines, and increased monocyte adhesion.

Our data suggest that the activation of senescent, pro-inflammatory, and chemoattractant gene expression promotes the progression of acute stress to endothelial dysfunction. The persistence of “protective” factors (i.e., resistance to apoptosis, cell cycle arrest, and decreased mitochondrial pro-apoptotic signaling) under significant oxidative stress shields dysfunctional ECs from being cleared, thus contributing to the chronic stress states of vascular diseases.

### HMOX1: A Master Regulator

While HMOX1 has been shown to influence many cellular functions in the past, these studies span many different stress conditions and cell types. Thus, they mostly provide a “snapshot” of HMOX1 regulated cellular function at a fixed time point (Gozzelino et al., 2010; Kim et al., 2011). Given that 51% of genes switch their direction of expression upon HP treatment in HMOX1-deficient cells (Figure 4C), it is clear that HMOX1 dramatically affects gene expression, highlighting its substantial influence on the endothelial OSR. To identify novel mechanisms of HMOX1 function, we identified switching genes that had not previously been associated with HMOX1 at each of the enriched locations (i.e., nucleus, mitochondria, and the cell surface; Figure 4D).

At the cell surface, HP induced EGFR and IGF1R were significantly dampened in HMOX1 deficient cells (Supplementary Figure 6). Consistent with our findings, it has been reported that the addition of HP increases the gene expression and protein concentration of EGFR (in murine melanoma cells) and IGF1R (in VSMCs; Du et al., 2001; Hyoudou et al., 2006). In addition, studies indicating the increased apoptosis of ECs upon EGFR inhibition (Bruns et al., 2000) support our finding that HMOX1-deficient cells treated with HP resulting in decreased EGFR expression and increased cell death (Figure 3C). Similarly, low IGF-1 is associated with increased endothelial apoptosis/dysfunction and cardiovascular risk (Conti et al., 2004). Additionally, it has been shown that growth factor signaling results in the phosphorylation of pro-apoptotic proteins, protecting the cell from death (Winn and Harlan, 2005). Taken together, the expression of EGFR and IGF1R (that informs the signaling flux through these receptors) influences decisions of cell fate in ECs. As such, targeted studies exploring the mechanism by which HMOX1 influences these receptors and the functional consequence of those changes could illuminate potential therapeutic targets for endothelial dysfunction and vascular diseases.

We and others have shown that HP treatment in HMOX1-competent HUVECs causes the downregulation of mitochondrial encoded ETC subunits (Karnewar et al., 2016); interestingly, we show that this is reversed in HMOX1 deficient HUVECs (Supplementary Figure 7). This reversal suggests that HMOX1 protects against mito-ROS mediated cell death by inhibiting expression or protein assembly of the ETC. Further, our characterization of mitochondrial respiration in HMOX1-competent and -deficient HUVECs suggests that the transcriptional changes associated with mitochondrial mRNA and protein processing may have lasting functional consequences. Indeed, our functional analysis of mitochondrial respiration indicates that consistent with the ETC subunit gene expression, the mitochondrial respiratory reserve capacity is significantly reduced in HP treated cells (Figure 5D and Supplementary Figure 8), making them vulnerable to metabolic stress and cell death. HMOX1 KD significantly restores the reserve capacity at the cost of increased proton leak with a marginal gain of ATP production (not statistically significant). However, continued proton leak could lead to mitochondrial membrane depolarization and mitopathy. Previous studies have shown that mitochondrial HMOX1 affects the flux through the ETC by regulating the availability of free heme and CO (Zuckerbraun et al., 2007; Heinemann et al., 2008; Almeida et al., 2015; Figure 5B).

Upon HP treatment, decreased expression of ETC subunits and down-regulation of post-transcriptional and post-translational regulators are consistent with decreased mitochondrial activity and mitochondrial-ROS mediated pro-death and pro-inflammatory processes. However, in HMOX1 deficiency, this protective mechanism is lost, and increased mitochondrial ROS likely promote dysfunction and death. Thus, while the role of HMOX1 in maintaining the redox potential of the mitochondria has been observed, our work provides novel mechanisms for therapeutic intervention to address dysfunctional ECs in vascular disease.

Given HMOX1’s entry and localization to the nucleus, its potential role in regulating transcription has been suggested (Lin et al., 2007; Biswas et al., 2014; Dulak and Jozkowicz, 2014). However, the role of HMOX1 in epigenetic regulation is less clear. We show that HMOX1 KD alters the expression of many histone modification genes and lnc-RNAs (Figures 5E,F). While the role of oxidative stress-mediated histone modification influencing HMOX1 expression is well characterized (Chervona and Costa, 2012), the only other study that has shown the role of HMOX1 in mediating histone modification genes’ expression (Ciesla et al., 2016). Furthermore, lnc-RNAs have been implicated in various aspects of the OSR (Huang et al., 2016; Kim et al., 2017). These novel aspects suggest that HMOX1-regulated endothelial OSR can influence transcriptional control and epigenetic modulations, thereby influencing a variety of cellular processes. However, their regulation by HMOX1 is yet to be explored.

In summary, our longitudinal analysis of the OS response in EC has elucidated dynamical mechanisms invoked, leading to cell fate decisions. As shown in literature extensively and based on our studies, ECs go toward cell death through apoptotic and necrotic mechanisms at higher OS levels. At low doses, the cells use recovery mechanisms, including redox enzymes involved in removing free radical species, induction of DNA repair mechanisms through transcription, and cell-protective mechanisms at the interface of genotoxic and metabolic stress. At a critical dose between cell recovery and death lies the mechanisms that poise the cell toward recovery or death. Our temporal studies of response at this dose point to many cellular responses, including several cell-protective mechanisms that suspend the cell at the interesting junction between cell survival and death. Most importantly, our study reveals a master regulator, HMOX1, which appears to play a critical role in regulating the spatiotemporal response in EC to OS. Whether the novel mechanisms of HMOX1 presented here occur either by decoupling its enzymatic and non-enzymatic activities or as a combination of the two requires further analysis. Our study also points to interesting avenues for designing therapeutics to deal with OS response, vital for vascular dysfunction and disease.

## Data Availability Statement

The data presented in the study are deposited in the GEO repository, accession number GSE72991.

## Author Contributions

SRg carried out the systems analyses of the longitudinal OSR in endothelial cells, conducted the knockdown experiments, analyzed HMOX1 knockdown data, and wrote the first draft of the manuscript along with SRm. SRm performed the dose-response experiments and functional validation of the time-course model, contributed to the corresponding Methods sections, and participated in the study design. EK helped with the time course transcriptomics and nextgen sequencing experiments, carried out the transcriptional data analysis, and contributed to the corresponding methods. YM helped optimize the Mito Stress Test assay and participated in data discussions on the mitochondrial assay. RL participated in the study design. ZC participated in the design of validation experiments with mitochondria assays. SS conceived the study, participated in its design, helped develop mechanistic models, revised the manuscript, and supervised the entire project. All authors were involved in editing and approving the manuscript.

## Author Disclaimer

The content is solely the responsibility of the authors (SS and ZC) and does not necessarily represent the official views of the National Institutes of Health.

## Conflict of Interest

The authors declare that the research was conducted in the absence of any commercial or financial relationships that could be construed as a potential conflict of interest.

## Publisher’s Note

All claims expressed in this article are solely those of the authors and do not necessarily represent those of their affiliated organizations, or those of the publisher, the editors and the reviewers. Any product that may be evaluated in this article, or claim that may be made by its manufacturer, is not guaranteed or endorsed by the publisher.
