# Supplementary material for: Heme Oxygenase-1 at the Nexus of Endothelial Cell Fate Decision Under Oxidative Stress
Source: Front Cell Dev Biol. 2021 Sep 14;9:702974. doi: 10.3389/fcell.2021.702974 (PMC8476872; doi:10.3389/fcell.2021.702974)
Supplement: Supplementary file 1 [file Data_Sheet_1.pdf]

Supplementary Material

Heme Oxygenase-1 at the Nexus of Endothelial Cell Fate Decision Under Oxidative Stress

Sindhushree Raghunandan[1†], Srinivasan Ramachandran[1†], Eugene Ke[1], Yifei Miao[5], Ratnesh Lal[$,1,2], Zhen Chen[$,5], Shankar Subramaniam[$*,1,3,4]

[1]Department of Bioengineering, University of California, San Diego, 9500 Gilman Drive, La Jolla, CA, USA

[2]Department of Mechanical and Aerospace Engineering, University of California, San Diego, 9500 Gilman Drive, La Jolla, CA, USA

[3]Department of Computer Science and Engineering, University of California, San Diego, 9500 Gilman Drive, La Jolla, CA, USA

[4]Department of Cellular and Molecular Medicine, University of California, San Diego, 9500 Gilman Drive, La Jolla, CA, USA

[5]Department of Diabetes Complications and Metabolism, City of Hope, 1500 E. Duarte Rd, Duarte, CA, USA

[†]These authors contributed equally to this work and share first authorship

[$]These authors share last authorship

***Correspondence:**
Shankar Subramaniam, 9500 Gilman Drive, La Jolla, CA, 92093-0427, (858) 822-0986, Fax: (858) 822-3752, shankar@ucsd.edu

Keywords: Oxidative stress, Reactive oxygen species, ROS, Hydrogen peroxide, Heme oxygenase-1, HMOX1, Endothelial cell, HUVEC.

1.1 Supplementary Figures

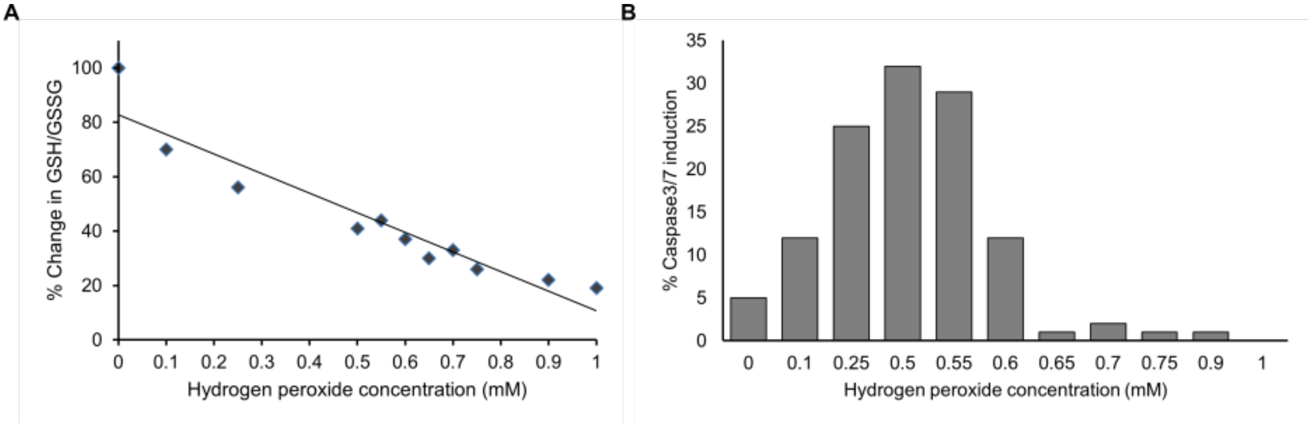

Supplementary Figure 1. The trends of anti-oxidant capacity and caspase3/7 activity as a function of HP dose. A) GSH/GSSG ratio was measured as an indicator of redox status and reported as a percent change to untreated samples at 12 hrs. Two biological experiments (n=2) each had three technical replicates. A negative trend line indicates decrease in cellular reduction capacity with increasing HP concentrations. B) Caspase 3 and 7 were measured as an indicator of apoptotic status. Caspase activity is reported as the percent of positive control (10 µM staurosporine) at 12 hours post-treatment. Two biological experiments (n=2) each had three technical replicates.

A

	Replicate 1		Replicate 2	
	Total read#	Uniquely mapped read%	Total read#	Uniquely mapped read%
**control**	22088557	83.9122	23782479	84.0059
**1h**	19739474	83.7663	22057285	83.6304
**2h**	19386750	84.0874	24870187	82.984
**4h**	14557529	84.2354	22377555	82.2981
**6h**	15434799	84.6441	17578932	83.9786
**8h**	17224818	83.691	23413820	84.0869
**10h**	17626853	83.3105	16690048	85.1201
**12h**	17909366	84.8559	18843315	85.2262
**14h**	20310499	86.1189	20790570	85.5375
**16h**	15039794	82.271	18047435	84.5686

B

	Replicate 1		Replicate 2	
	Total read#	Uniquely mapped read%	Total read#	Uniquely mapped read%
**Scrambled-NT**	23464850	87.6069	18695249	68.7756
**Scrambled + H2O2**	15574457	88.4159	27920902	88.1699
**siHMOX1 + H2O2**	26549836	87.5914	25304702	87.56

C

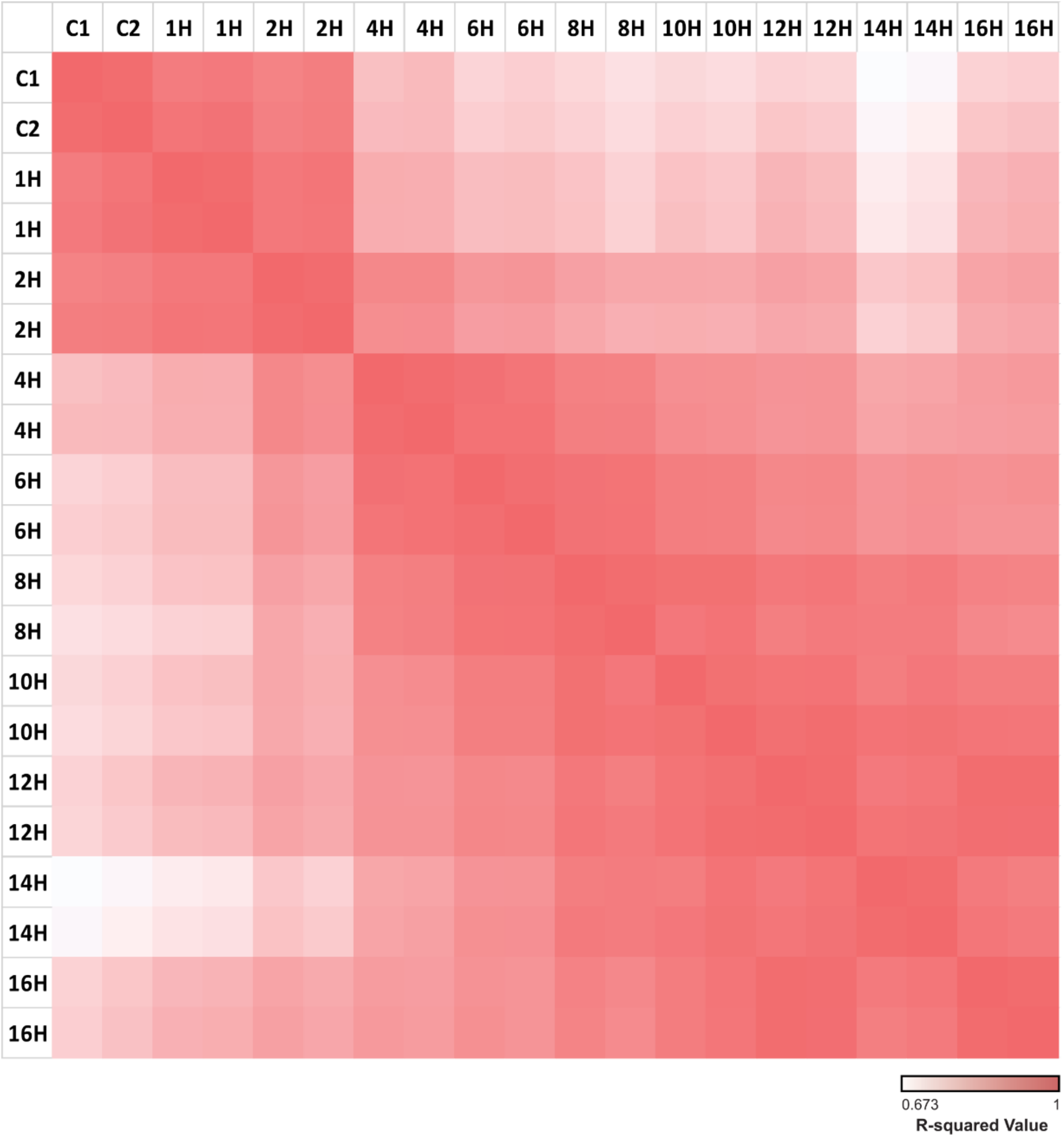

Supplementary Figure 2. **Summary of data quality (read, alignment, and replicate quality)** A) All samples in the time-series experiment had ~20 million reads; the average uniquely mapped alignment percentage was ~84·11%. B) All samples in the knockdown experiment had ~23 million reads; the average uniquely mapped alignment percentage was ~85% C) The R^2 value was calculated for all pairwise comparisons between samples in the time-series experiment using raw gene counts from the OSA2 output. The legend indicating the range of R^2 values is shown at the bottom left of the heatmap.

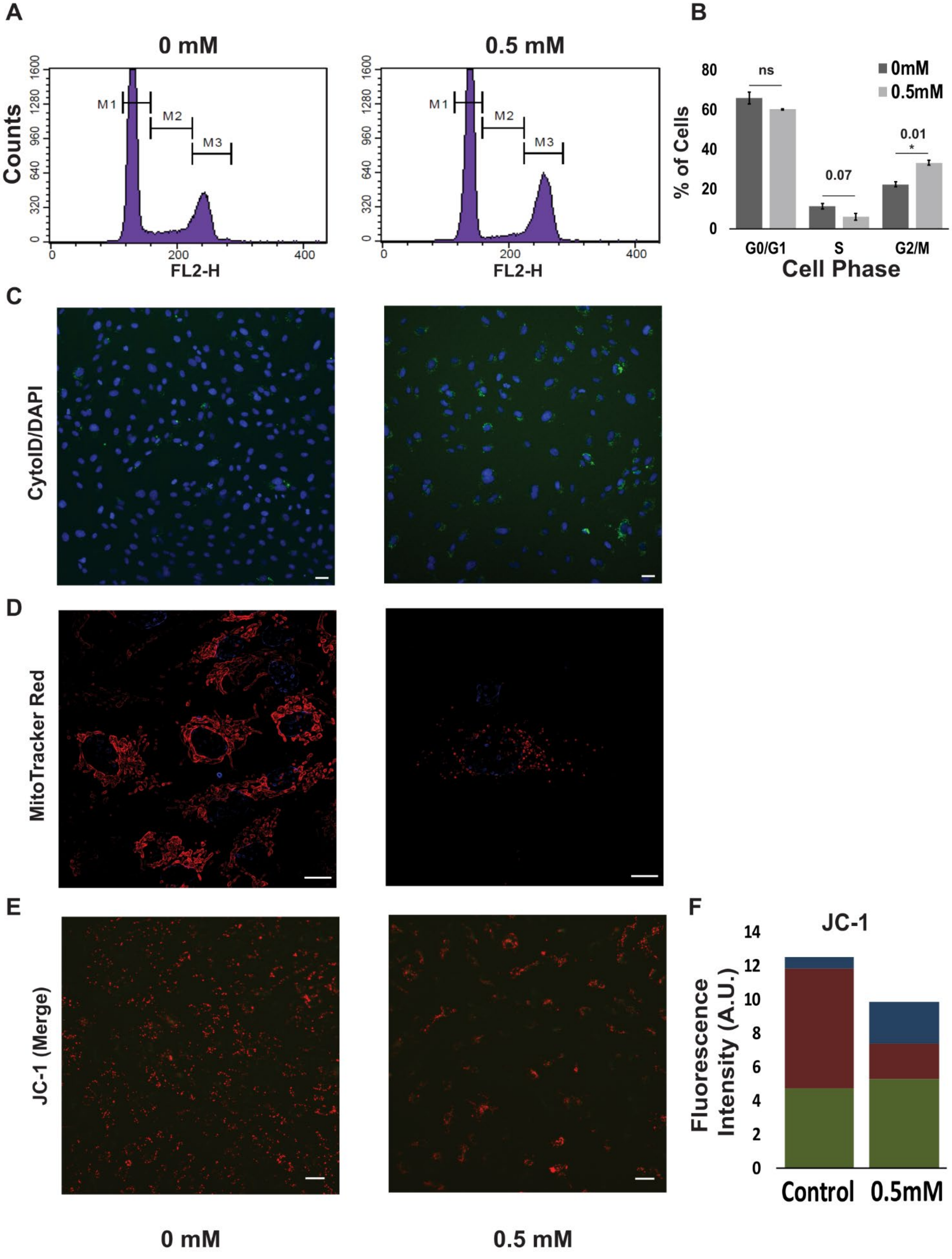
A
0 mM
0.5 mM
Counts
FL2-H
M1
M2
M3
B
% of Cells
0mM
0.5mM
ns
0.07
0.01
*
G0/G1
S
G2/M
Cell Phase
C
CytoID/DAPI
D
MitoTracker Red
E
JC-1 (Merge)
F
JC-1
Fluorescence Intensity (A.U.)
Control
0.5mM
0 mM
0.5 mM

Supplementary Figure 3. Phenotypic trends of Temporal Model of OSR. HUVECs treated with 0 or 0·5 mM HP, and phenotypic measurements were taken to verify the transcriptomic changes' functional consequences in response to oxidative stress. A & B) Cell cycle analysis by flow cytometry. Ten thousand events were collected for each sample, and their histograms were plotted. The first peak represents the distribution of cells in the G0/G1 phase (M1), the second peak represents cells in the S phase (M2), and the third peak represents cells in the G2/M phase (M3). The average percentage of cells in G0/G1, S, and G2/M phases in control (dark gray) and 0·5 mM (light gray) treated samples; error bars indicate standard deviation of the average calculated across two biological repeats of the experiment. P-values labeled above each group; $p < 0{\cdot}05$ indicated by *. C) Autophagy detection Cyto-ID. Autophagic vacuoles (green) were imaged with excitation at 463 nm and emission at 534 nm. Scale bar: 10 µm. Two independent biological experiments each had two technical replicates. D) Mitochondrial network architecture imaged with MitoTraker Deep Red FM dye. 3-5 random fields were chosen for 100x imaging. Images were analyzed in ImageJ software. Edge detection techniques (Laplacian of Gaussian) were applied to visualize the mitochondrial network, distribution, and size. The maximum intensity projection was applied to determine the total intensity of the mitochondrial stain. The image on the left shows untreated cells, and the image on the right shows 0·5 mM HP treated cells. Scale bar: 5 µm. Two independent biological experiments each had two technical replicates. E & F) Mitochondrial membrane potential was measured with potentiostatic dye JC-1 (5 µM). For JC-1 monomers and aggregates, excitation and emission wavelengths of 485/535 and 560/595 nm, respectively, were used. Images were analyzed in ImageJ using a macro that automatically segments and quantifies green and red fluorescence. Mitochondrial membrane depolarization is indicated by green fluorescent monomers (green section of the bar graph), and healthy mitochondria are indicated by red fluorescent aggregates (red section of the bar graph). Ratios of monomers/aggregates (green/red ratio, the blue section of the bar graph) indicated the overall health of mitochondria in control and treated HUVECs. Scale bar: 10 µm. Two independent biological experiments each had two technical replicates.

Log2(Fold-Change)

		1h	2h	4h	6h	8h	10h	12h	14h	16h
	GeneName	l2fc_1h	l2fc_2h	l2fc_4h	l2fc_6h	l2fc_8h	l2fc_10h	l2fc_12h	l2fc_14h	l2fc_16h
Cell Cycle	CCNE1	-0.105	-0.351	-1.329	-1.184	-1.505	-1.296	-1.249	-1.368	-0.908
	MCM2	0.057	-0.032	-0.300	-0.801	-1.352	-1.652	-1.774	-2.090	-2.087
	MCM3	0.002	-0.051	-0.212	-0.666	-1.225	-1.572	-1.456	-1.400	-1.334
	MCM4	-0.034	-0.155	-0.457	-1.110	-1.624	-2.317	-2.058	-2.396	-2.058
	MCM5	0.073	-0.086	-0.188	-0.648	-1.136	-1.519	-1.758	-1.931	-1.803
	CCNB1	-0.081	-0.376	-1.936	-1.883	-2.110	-2.663	-2.823	-3.427	-2.678
	CCNB2	0.016	-0.026	-0.644	-1.159	-1.770	-2.245	-2.628	-2.858	-2.531
	CDK1	-0.122	0.102	-0.345	-1.417	-2.025	-2.917	-2.665	-3.540	-2.361
	CDC20	0.105	-0.124	-0.942	-1.450	-2.082	-2.518	-3.059	-3.491	-2.835
	ANAPC4	-0.104	-0.110	-0.603	-1.344	-0.554	-0.230	-0.162	-0.043	-0.163
DNA Replication & Repair	DNA2	-0.063	0.152	-0.564	-1.508	-1.411	-1.664	-1.709	-1.939	-1.687
	FEN1	-0.102	-0.478	-0.920	-1.493	-2.064	-2.326	-2.154	-2.757	-2.195
	LIG1	0.074	-0.062	-0.190	-0.301	-0.497	-0.489	-0.667	-0.827	-0.733
	MCM2	0.057	-0.032	-0.300	-0.801	-1.352	-1.652	-1.774	-2.090	-2.087
	MCM3	0.002	-0.051	-0.212	-0.666	-1.225	-1.572	-1.456	-1.400	-1.334
	MCM4	-0.034	-0.155	-0.457	-1.110	-1.624	-2.317	-2.058	-2.396	-2.058
	MCM5	0.073	-0.086	-0.188	-0.648	-1.136	-1.519	-1.758	-1.931	-1.803
	MCM6	-0.014	-0.072	-0.563	-1.297	-2.077	-2.391	-1.929	-2.053	-1.786
	MCM7	0.061	0.034	0.246	0.060	-0.564	-1.142	-1.649	-1.888	-1.980
	POLA1	-0.048	-0.182	-0.444	-0.644	-1.143	-1.683	-1.396	-1.605	-1.509
	POLA2	0.007	-0.098	-0.517	-0.881	-1.465	-1.602	-1.497	-1.844	-1.645
	POLE	-0.029	-0.339	-0.415	-0.647	-0.561	-0.680	-0.783	-1.023	-0.954
	POLE2	-0.253	-0.332	-0.757	-1.960	-2.503	-2.690	-2.285	-2.290	-2.095
	POLE3	-0.011	0.012	0.049	0.045	-0.052	-0.508	-0.679	-0.724	-0.806
	PRIM1	-0.211	0.217	0.060	-0.908	-1.382	-1.668	-1.695	-1.615	-1.803
	RFC2	0.019	0.010	-0.212	-0.705	-1.247	-1.480	-1.381	-1.561	-1.338
	RFC3	-0.040	0.061	0.004	-0.258	-0.618	-1.364	-1.477	-1.778	-1.387
	RFC4	-0.153	0.056	-0.556	-1.459	-1.800	-1.772	-1.666	-1.559	-1.636
	RFC5	-0.081	-0.065	-0.492	-0.923	-1.425	-1.521	-1.695	-1.786	-1.621
	RNASEH2A	0.133	-0.072	-0.118	-0.324	-0.990	-1.173	-1.090	-1.089	-1.211
	RNASEH2B	-0.038	0.094	-0.184	-0.546	-0.890	-0.896	-0.718	-0.663	-0.718
	RNASEH2C	-0.013	-0.135	0.118	-0.291	-0.494	-0.407	-0.730	-0.722	-0.591
	RPA1	-0.009	-0.061	-0.314	-0.583	-0.906	-1.042	-0.790	-0.782	-0.800
	RPA2	0.070	0.086	-0.193	-0.531	-0.643	-0.897	-0.957	-1.029	-0.904
	RPA3	0.004	0.101	0.156	-0.073	-0.592	-1.096	-1.182	-1.436	-1.159
	CHEK1	0.034	0.102	-0.411	-0.922	-1.244	-1.335	-1.201	-1.422	-1.176
	CHEK2	-0.008	0.112	-0.109	-1.121	-1.578	-1.696	-1.158	-1.346	-1.016
	ATM	-0.132	-0.228	-0.274	-0.849	-0.642	-0.591	0.003	0.021	0.265
	ATR	-0.104	-0.307	-0.814	-0.788	-0.330	-0.430	0.037	-0.217	-0.049
Mitochondrial Activity	MT-ND4	-0.511	-0.215	-0.521	-0.749	-0.896	-1.345	-1.866	-1.542	-2.181
	MT-ND4L	-1.107	-0.246	-0.314	-0.711	-0.488	-1.041	-1.467	-1.105	-1.784
	MT-CO2	-0.634	-0.582	-1.091	-1.587	-1.989	-2.559	-2.717	-2.989	-2.480
	MT-CO3	-0.599	-0.733	-1.255	-1.797	-2.131	-2.636	-2.974	-3.067	-2.813
	MT-ND2	-0.555	-0.187	-0.604	-0.804	-0.900	-1.249	-1.328	-1.336	-1.111
	MT-ND3	-0.636	-0.731	-1.338	-1.881	-1.958	-2.416	-2.601	-2.637	-2.009
	MT-ATP8	-0.771	-0.266	-0.570	-1.076	-0.937	-1.633	-2.057	-1.702	-2.362
	MT-ATP6	-0.494	-0.032	-0.408	-0.716	-0.876	-1.345	-1.854	-1.548	-2.145
TGFb Signaling	SMURF1	0.144	0.135	0.607	1.168	0.840	0.478	0.382	0.472	0.416
	INHBC	0.132	2.773	3.894	2.115	1.260	0.911	0.377	0.092	NA
	SMAD6	0.107	2.376	4.640	2.697	0.482	-0.833	-0.505	-0.942	-0.659
	SMAD7	1.738	4.446	4.101	3.036	2.044	1.821	1.481	1.529	0.207
Apoptosis	BAK1	-0.053	-0.388	0.050	0.732	0.963	0.873	0.637	0.609	0.358
	BAX	0.065	0.169	0.724	0.714	0.511	0.385	0.248	0.196	0.262
	DDIT3	1.539	3.943	4.219	2.899	2.429	2.281	2.220	3.060	1.650
	NR4A1	2.473	4.083	3.178	2.176	1.408	1.678	1.451	1.524	0.947
	BRCA1	-0.145	-0.018	-0.063	-0.797	-1.505	-1.955	-1.672	-1.891	-1.635
	BIRC2	0.027	0.706	1.321	1.260	1.074	0.455	0.175	0.359	0.231
	BIRC3	-0.345	2.013	3.212	3.868	3.917	2.937	1.768	1.978	1.305
	CASP7	-0.041	0.076	-0.482	-0.764	-0.424	-0.172	-0.153	-0.278	-0.158
	CASP8	-0.137	-0.023	-0.400	-0.893	-0.386	-0.014	0.162	0.027	0.026
	TRADD	-0.045	-0.402	-0.572	-0.646	-0.360	0.094	0.159	0.224	0.274
	FADD	-0.190	-0.715	-0.753	-0.488	-0.335	-0.345	-0.555	-0.431	-0.419
	FAS	0.036	0.896	2.965	3.253	3.235	2.694	2.596	2.367	1.993
	BCL2L1	-0.058	-0.208	0.610	0.679	0.576	0.662	0.640	0.676	0.611
	BCL2L2	0.172	0.430	0.863	0.627	0.495	0.465	0.310	0.447	0.257
TNF/Cytokine Signaling	C1S	0.018	3.375	4.797	4.411	3.704	3.043	1.229	1.990	0.698
	C3	0.009	1.405	3.273	2.658	2.100	1.353	0.800	1.331	0.751
	C9	-0.116	1.802	4.263	3.178	1.937	0.850	0.217	0.415	0.137
	C4A	-0.163	0.711	1.949	1.949	0.798	0.812	1.201	1.200	1.019
	IL11	0.369	3.848	5.210	4.614	3.797	3.108	1.774	1.756	0.728
	IL1A	0.406	1.340	2.823	2.213	1.293	0.829	0.612	1.824	0.579
	IL1B	0.539	0.676	1.155	0.534	0.609	1.021	1.027	1.282	0.324
	CXCL2	-1.262	-1.929	-1.724	-1.277	-2.119	-1.597	-1.045	-0.297	-0.642
	CSF1	0.213	0.700	1.476	1.995	2.155	2.132	1.778	1.788	1.103
	LIF	0.210	2.922	4.842	5.298	5.192	4.887	3.823	4.181	2.858
	IFNE	0.266	1.878	3.122	1.968	1.852	0.822	0.456	0.722	0.721
	TNFSF13	0.521	1.074	1.372	0.845	0.396	0.802	0.393	0.158	0.386
	LTA	0.328	2.182	2.511	1.917	1.474	1.129	0.321	0.326	-0.236
	TNFRSF1A	-0.062	-0.502	-0.397	0.329	0.365	0.367	0.295	0.404	0.160
	TNFRSF10A	0.115	0.445	1.376	1.843	1.808	1.465	1.098	1.200	0.551
	TNFRSF10B	0.061	0.514	1.538	1.725	1.472	0.981	0.646	0.782	0.526
	TNFSF10	0.124	-0.052	-0.771	-2.021	-2.211	-2.166	-1.694	-1.994	-0.244
Cell Adhesion	ICAM1	0.146	1.224	1.590	1.656	1.723	1.709	1.058	2.078	1.056
	SELE	0.270	2.150	3.606	2.554	0.851	-0.911	-1.178	-0.445	0.263
NFKB Signaling	NFKBIA	0.622	1.155	1.427	1.496	0.883	0.433	0.363	0.819	0.760
	NFKBIE	0.175	0.686	1.147	1.331	0.951	0.452	0.398	0.572	0.585
	RELA	0.134	0.178	0.126	0.641	0.557	0.472	0.153	0.165	0.094
	RELB	-0.146	0.252	1.358	2.242	2.226	1.991	1.430	1.770	1.437
	NFKB1	-0.117	-0.111	-0.019	0.645	0.539	0.271	0.024	0.159	-0.041
	NFKB2	-0.084	0.188	0.376	1.224	1.459	1.396	0.935	1.139	0.829
Cell-Cell/Cell-Matrix Interaction	CDH5	0.004	-0.120	0.096	0.193	0.111	0.208	0.277	0.244	0.403
	VEGFA	0.302	1.927	2.677	2.368	2.109	1.788	1.239	1.663	0.839
Autophagy	MAP1LC3A	0.155	-0.007	0.628	0.750	0.547	0.671	0.730	0.799	0.799
	MAP1LC3B	0.079	0.676	1.279	1.373	1.212	0.748	0.596	0.930	0.516
	GABARAPL1	0.087	0.558	1.061	1.456	1.419	0.974	0.303	0.532	0.199
	ULK1	0.117	0.148	0.493	0.974	0.950	0.636	0.225	0.165	0.279
	ATG4D	0.105	0.023	0.107	0.607	0.755	0.798	0.308	0.520	0.180
	AMBRA1	0.123	-0.233	0.360	0.820	0.437	0.336	0.013	0.108	0.051
	SQSTM1	-0.002	0.477	0.906	1.777	2.026	1.882	1.283	1.718	1.233
	PIK3C3	-0.131	-0.298	-0.035	0.441	0.724	0.458	0.407	0.499	0.523
Lysosome	DRAM1	-0.1388	0.1203	1.2697	1.4545	1.2879	0.91899	0.86269	0.81745	0.70878
	FUCA1	0.400	0.154	0.661	0.937	1.115	1.292	1.521	1.624	1.660
	DNASE2	0.247	0.101	0.464	0.614	0.722	0.877	0.875	1.077	0.989
	ASAH1	-0.021	0.135	0.398	0.390	0.488	0.481	0.664	0.870	1.030
	LAMP3	-0.099	-0.064	0.005	0.331	0.568	0.513	0.653	1.238	0.896
	PSAP	0.003	-0.020	0.318	0.469	0.425	0.488	0.435	0.589	0.597
	MANBA	0.107	1.075	0.962	0.386	0.571	0.238	0.428	0.451	0.732
	IDS	0.116	-0.233	0.048	-0.021	0.240	0.225	0.500	0.277	-0.149
	MFSD8	0.060	0.108	-0.174	-0.127	0.181	0.355	0.578	0.565	0.504
	ENTPD4	-0.116	-0.046	-0.225	-0.088	0.308	0.410	0.447	0.346	0.474
	ABCA2	0.035	0.048	0.686	0.024	-0.043	0.068	0.252	0.164	0.628
	HEXA	0.067	0.050	0.369	0.376	0.187	0.254	0.263	0.327	0.485
	CLN5	-0.067	0.086	0.084	-0.104	-0.082	-0.069	0.284	0.363	0.537
	GALC	0.000	0.116	0.026	-0.092	-0.024	-0.130	0.264	0.273	0.473
	LAMP2	-0.045	0.106	0.281	0.123	0.174	0.058	0.271	0.208	0.392
	GALNS	0.006	-0.041	0.188	0.027	-0.025	0.043	0.063	0.162	0.391
Antioxidant Response	NOS3	0.126	-0.169	-0.802	-1.024	-0.744	-0.165	0.067	-0.327	-0.263
	CAT	0.039	0.015	-0.304	-0.643	-0.457	-0.478	-0.235	-0.228	0.076
	PRDX3	0.002	0.105	0.144	-0.257	-0.433	-0.652	-0.413	-0.546	-0.308
	TXNRD2	-0.042	-0.197	0.100	-0.212	-0.382	-0.309	-0.378	-0.396	-0.187
Stress Response TFs	KLF2	1.411	1.605	-0.736	-0.015	-0.291	-0.261	-0.660	-0.444	-0.942
	KLF4	4.093	4.497	2.251	1.430	1.528	1.648	1.104	2.119	0.707
Thrombosis	THBD	0.291	0.866	0.743	0.221	0.821	1.174	1.068	1.212	0.601

P-value

		1h	2h	4h	6h	8h	10h	12h	14h	16h
	GeneName	pval_1h	pval_2h	pval_4h	pval_6h	pval_8h	pval_10h	pval_12h	pval_14h	pval_16h
Cell Cycle	CCNE1	5.11E-01	1.70E-02	5.36E-14	5.96E-11	9.82E-17	2.86E-15	2.49E-13	4.55E-13	2.16E-10
	MCM2	5.10E-01	5.77E-01	8.13E-07	1.21E-23	5.36E-45	3.96E-18	5.51E-46	2.28E-143	2.80E-61
	MCM3	9.69E-01	3.83E-01	6.15E-06	2.59E-22	1.18E-39	5.64E-128	3.01E-98	2.82E-126	6.16E-175
	MCM4	5.13E-01	1.14E-04	6.65E-23	1.06E-62	4.64E-69	2.36E-232	1.11E-49	1.06E-278	0.00E+00
	MCM5	4.83E-01	3.83E-02	4.49E-04	1.16E-31	3.77E-65	1.34E-33	6.45E-128	2.17E-156	2.09E-205
	CCNB1	1.66E-01	2.53E-10	2.15E-131	1.17E-126	8.74E-173	9.24E-243	2.49E-255	0.00E+00	1.02E-240
	CCNB2	7.89E-01	6.21E-01	1.73E-24	6.86E-14	3.33E-52	3.34E-89	1.64E-174	2.12E-178	9.19E-224
	CDK1	5.99E-02	1.04E-01	1.90E-02	4.79E-21	3.07E-61	1.21E-158	2.30E-157	6.34E-242	1.48E-212
	CDC20	1.08E-01	7.37E-03	1.33E-81	1.95E-52	3.55E-69	1.04E-44	3.18E-144	0.00E+00	1.64E-291
	ANAPC4	5.06E-01	2.67E-01	3.68E-05	2.85E-09	3.94E-06	2.96E-01	1.63E-01	7.38E-01	1.05E-01
DNA Replication & Repair	DNA2	6.45E-01	1.81E-01	2.84E-04	1.95E-17	1.99E-15	1.87E-19	7.51E-25	1.16E-26	1.66E-30
	FEN1	2.38E-01	8.26E-21	2.80E-51	5.42E-70	2.37E-33	2.35E-182	8.58E-53	1.10E-215	1.46E-216
	LIG1	4.10E-01	5.09E-01	6.96E-02	6.19E-03	1.55E-06	2.67E-05	8.85E-12	2.02E-15	1.67E-08
	MCM2	5.10E-01	5.77E-01	8.13E-07	1.21E-23	5.36E-45	3.96E-18	5.51E-46	2.28E-143	2.80E-61
	MCM3	9.69E-01	3.83E-01	6.15E-06	2.59E-22	1.18E-39	5.64E-128	3.01E-98	2.82E-126	6.16E-175
	MCM4	5.13E-01	1.14E-04	6.65E-23	1.06E-62	4.64E-69	2.36E-232	1.11E-49	1.06E-278	0.00E+00
	MCM5	4.83E-01	3.83E-02	4.49E-04	1.16E-31	3.77E-65	1.34E-33	6.45E-128	2.17E-156	2.09E-205
	MCM6	8.29E-01	2.36E-01	2.25E-21	2.96E-74	1.13E-37	8.50E-206	2.66E-91	1.70E-171	5.45E-201
	MCM7	2.01E-01	4.98E-01	5.98E-08	2.12E-01	2.49E-10	7.55E-18	1.44E-194	1.22E-284	9.47E-236
	POLA1	5.72E-01	2.99E-02	1.18E-05	4.12E-05	1.33E-26	3.23E-45	1.89E-39	2.50E-45	2.76E-56
	POLA2	9.43E-01	4.00E-01	9.46E-06	2.95E-07	5.47E-32	3.42E-21	1.40E-32	6.51E-37	3.95E-47
	POLE	8.08E-01	1.12E-02	1.43E-03	1.60E-06	1.79E-05	6.86E-07	1.60E-10	2.03E-15	2.58E-14
	POLE2	5.05E-02	2.88E-03	2.09E-08	1.11E-25	1.26E-28	9.96E-51	3.40E-38	1.47E-16	1.44E-43
	POLE3	8.83E-01	8.09E-01	3.30E-01	4.66E-01	6.77E-01	9.75E-13	8.31E-27	5.67E-33	1.27E-42
	PRIM1	1.01E-01	5.25E-02	6.62E-01	1.23E-08	1.91E-12	1.25E-25	6.71E-23	8.53E-15	1.49E-26
	RFC2	8.47E-01	9.00E-01	1.66E-02	5.81E-11	5.18E-27	7.93E-39	1.82E-39	5.96E-42	4.18E-32
	RFC3	6.82E-01	5.49E-01	9.76E-01	1.07E-01	2.52E-04	9.88E-32	5.12E-37	8.58E-44	2.34E-29
	RFC4	1.27E-01	5.20E-01	3.32E-07	1.11E-27	3.58E-38	1.15E-42	3.14E-35	2.14E-32	3.93E-44
	RFC5	3.89E-01	5.01E-01	3.35E-06	5.79E-15	2.88E-13	5.96E-34	5.93E-41	4.15E-42	1.55E-39
	RNASEH2A	2.11E-01	4.83E-01	2.69E-01	7.11E-03	2.12E-09	1.23E-15	8.29E-10	1.62E-15	3.25E-14
	RNASEH2B	7.42E-01	4.76E-01	1.22E-01	1.34E-04	1.18E-05	1.22E-07	1.07E-07	4.13E-07	1.25E-09
	RNASEH2C	8.87E-01	1.20E-01	1.76E-01	5.13E-03	6.55E-05	1.24E-02	1.73E-12	3.27E-11	2.58E-11
	RPA1	8.76E-01	2.96E-01	5.40E-09	8.96E-24	5.92E-24	1.52E-68	4.58E-34	2.79E-43	1.05E-52
	RPA2	5.32E-01	4.51E-01	1.47E-01	3.95E-04	5.30E-06	4.82E-12	9.99E-13	5.43E-14	5.29E-14
	RPA3	9.77E-01	3.55E-01	1.62E-01	6.50E-01	4.33E-06	2.38E-10	8.94E-17	7.67E-21	1.55E-20
	CHEK1	6.73E-01	2.90E-01	1.50E-06	3.73E-18	2.04E-32	1.64E-33	3.95E-32	6.52E-40	5.69E-37
	CHEK2	9.52E-01	4.02E-01	4.43E-01	4.54E-10	2.16E-17	4.04E-22	9.91E-11	4.05E-10	9.76E-12
	ATM	4.04E-01	2.29E-01	1.33E-01	5.69E-04	3.67E-04	2.19E-02	9.87E-01	9.04E-01	1.59E-01
	ATR	3.04E-01	5.75E-03	5.50E-13	2.67E-10	9.35E-03	8.23E-04	7.57E-01	5.28E-02	6.79E-01
Mitochondrial Activity	MT-ND4	6.64E-05	1.57E-01	2.80E-03	3.51E-04	5.90E-11	4.14E-21	2.61E-30	4.33E-36	1.47E-55
	MT-ND4L	1.71E-22	3.43E-03	2.98E-02	2.93E-14	3.54E-05	6.83E-28	4.81E-37	1.57E-36	1.22E-53
	MT-CO2	7.55E-08	5.45E-06	8.08E-12	1.96E-26	1.64E-35	6.53E-74	1.03E-111	4.32E-141	1.30E-87
	MT-CO3	5.10E-07	6.21E-12	2.02E-21	2.03E-53	4.25E-44	2.05E-73	2.43E-179	1.38E-189	4.01E-118
	MT-ND2	9.06E-12	3.09E-02	1.27E-04	1.07E-08	3.30E-11	2.57E-29	2.52E-54	1.13E-55	2.83E-36
	MT-ND3	1.13E-08	9.52E-17	6.07E-39	5.82E-87	1.69E-30	2.67E-65	2.01E-79	6.39E-142	1.97E-120
	MT-ATP8	2.79E-13	2.32E-02	4.14E-03	4.31E-15	3.31E-06	7.31E-36	2.73E-48	6.28E-42	6.70E-79
	MT-ATP6	9.13E-08	7.50E-01	8.13E-03	1.26E-05	4.96E-13	9.67E-31	4.18E-49	4.45E-59	4.48E-68
TGFb Signaling	SMURF1	1.92E-01	3.52E-01	2.94E-08	7.76E-16	5.32E-13	2.07E-04	5.78E-04	9.41E-06	1.18E-05
	INHBC	3.69E-01	9.74E-10	1.08E-11	2.73E-04	1.43E-02	4.53E-02	2.17E-01	5.99E-01	NA
	SMAD6	6.53E-01	7.98E-39	1.71E-188	1.03E-34	1.06E-01	1.49E-02	8.21E-02	1.43E-03	2.04E-02
	SMAD7	2.98E-09	8.23E-93	5.46E-74	2.62E-33	2.15E-11	5.08E-11	1.43E-07	4.92E-07	5.15E-01
Apoptosis	BAK1	7.39E-01	1.40E-05	5.17E-01	2.74E-18	2.38E-33	7.32E-09	4.39E-14	6.70E-09	9.87E-08
	BAX	3.04E-01	2.45E-04	2.98E-23	3.77E-28	4.15E-14	6.57E-03	9.71E-07	4.57E-03	2.30E-10
	DDIT3	4.98E-11	2.45E-219	6.55E-246	1.82E-98	1.84E-40	6.32E-55	4.55E-46	1.82E-106	1.11E-22
	NR4A1	2.31E-18	8.61E-60	1.00E-53	1.07E-22	2.55E-07	8.12E-11	3.75E-10	2.86E-08	3.27E-05
	BRCA1	1.27E-01	9.02E-01	5.96E-01	2.63E-11	8.45E-18	1.55E-61	2.75E-44	2.12E-47	1.20E-47
	BIRC2	7.71E-01	1.32E-20	1.93E-37	1.22E-21	1.32E-21	3.29E-03	5.30E-02	5.25E-04	5.27E-04
	BIRC3	2.93E-01	2.16E-17	8.09E-44	1.84E-47	4.03E-54	6.45E-35	2.88E-09	2.16E-09	6.27E-07
	CASP7	6.48E-01	3.72E-01	3.00E-07	1.55E-11	1.91E-04	8.85E-02	1.09E-01	7.51E-03	7.23E-02
	CASP8	3.56E-01	8.70E-01	3.18E-02	2.27E-07	1.31E-02	9.21E-01	3.18E-01	8.60E-01	8.37E-01
	TRADD	7.46E-01	2.68E-03	2.12E-04	7.45E-03	3.32E-02	6.00E-01	2.74E-01	1.48E-01	2.74E-02
	FADD	1.07E-01	4.33E-12	2.58E-16	1.34E-05	7.52E-03	3.58E-04	3.69E-06	1.44E-04	1.01E-03
	FAS	8.48E-01	6.39E-09	6.63E-113	9.22E-85	1.76E-79	1.38E-53	1.82E-86	2.59E-46	1.29E-52
	BCL2L1	4.71E-01	5.61E-05	1.75E-29	2.64E-31	2.88E-30	2.47E-07	1.48E-30	3.48E-12	3.51E-43
	BCL2L2	1.20E-01	3.47E-04	1.81E-18	2.52E-08	4.41E-06	4.49E-05	3.65E-02	3.53E-05	2.75E-02
TNF/Cytokine Signaling	C1S	9.53E-01	5.55E-17	3.31E-25	9.24E-23	9.51E-17	4.44E-12	7.77E-03	2.80E-05	1.13E-01
	C3	9.70E-01	2.46E-03	3.01E-09	1.56E-06	6.75E-05	7.59E-03	7.27E-02	7.48E-03	7.73E-02
	C9	3.85E-01	1.03E-04	2.28E-16	1.55E-09	2.89E-04	8.40E-02	5.64E-01	3.47E-01	6.82E-01
	C4A	6.35E-01	2.12E-02	9.65E-10	3.93E-11	1.20E-01	7.60E-02	9.71E-05	2.08E-04	4.84E-04
	IL11	2.86E-01	4.07E-29	2.57E-44	3.61E-34	4.25E-21	1.25E-14	3.31E-05	1.21E-04	9.27E-02
	IL1A	1.23E-01	4.28E-08	3.94E-36	5.09E-15	5.13E-05	2.34E-03	9.29E-02	2.01E-13	1.24E-02
	IL1B	6.00E-02	9.79E-02	6.06E-02	2.99E-01	2.13E-01	3.92E-02	2.08E-02	8.81E-03	3.44E-01
	CXCL2	6.71E-08	5.72E-14	5.07E-10	5.07E-03	4.62E-11	1.44E-10	1.93E-03	2.29E-01	1.53E-03
	CSF1	2.15E-01	2.50E-07	1.35E-25	5.61E-47	5.06E-50	1.71E-38	1.87E-44	2.56E-32	4.36E-13
	LIF	5.12E-01	1.43E-14	1.85E-34	1.86E-45	2.12E-48	3.07E-45	2.48E-25	9.83E-30	5.32E-13
	IFNE	4.39E-01	1.13E-05	8.09E-12	8.44E-05	8.99E-05	1.02E-01	3.20E-01	1.42E-01	1.00E-01
	TNFSF13	6.72E-02	2.32E-06	4.07E-09	2.06E-03	2.32E-01	5.75E-03	1.64E-01	6.56E-01	2.17E-01
	LTA	3.52E-01	7.42E-11	2.85E-11	2.53E-06	1.26E-03	1.32E-02	4.51E-01	4.82E-01	5.86E-01
	TNFRSF1A	4.49E-01	1.99E-07	3.77E-05	3.84E-04	4.75E-06	5.76E-04	3.82E-04	3.18E-07	3.88E-02
	TNFRSF10A	3.50E-01	2.37E-05	4.22E-40	2.52E-53	1.07E-50	1.08E-42	2.30E-23	2.51E-28	6.06E-08
	TNFRSF10B	3.89E-01	7.10E-14	4.73E-187	1.12E-245	1.36E-104	2.64E-22	5.93E-48	4.03E-68	2.61E-25
	TNFSF10	5.55E-01	7.97E-01	3.62E-03	6.81E-12	2.95E-11	4.96E-16	2.46E-09	2.87E-11	2.88E-01
Cell Adhesion	ICAM1	4.02E-01	4.65E-27	4.53E-44	7.06E-39	1.22E-16	2.19E-18	2.59E-11	1.13E-75	2.05E-23
	SELE	3.59E-01	2.84E-22	2.78E-88	3.82E-27	4.99E-02	2.13E-02	9.72E-04	1.38E-01	2.63E-01
NFKB Signaling	NFKBIA	4.73E-07	7.86E-26	2.11E-52	1.02E-47	1.56E-13	1.72E-04	1.13E-03	1.29E-14	3.01E-16
	NFKBIE	2.62E-01	9.24E-09	1.15E-15	3.42E-21	1.60E-13	4.91E-04	3.88E-03	5.22E-05	2.78E-07
	RELA	1.98E-02	2.15E-02	2.75E-01	4.57E-06	8.99E-25	2.57E-05	1.59E-02	5.22E-02	1.34E-01
	RELB	4.15E-01	1.15E-01	3.93E-17	3.55E-41	4.30E-57	7.47E-22	1.38E-18	9.02E-25	1.66E-29
	NFKB1	1.04E-01	1.97E-01	7.69E-01	4.01E-07	2.06E-18	2.89E-04	7.08E-01	1.60E-02	4.99E-01
	NFKB2	4.87E-01	6.69E-02	4.75E-03	2.14E-30	1.82E-52	2.25E-13	2.10E-19	1.07E-27	1.37E-19
Cell-Cell/Cell-Matrix Interaction	CDH5	9.52E-01	2.21E-02	8.14E-02	4.34E-02	4.73E-02	2.47E-02	5.54E-09	7.58E-04	1.58E-22
	VEGFA	5.54E-02	2.25E-33	1.99E-168	1.04E-67	1.80E-79	2.20E-30	4.08E-07	5.53E-49	2.20E-11
Autophagy	MAP1LC3A	5.34E-01	9.78E-01	1.22E-02	4.05E-03	3.82E-02	5.81E-03	2.95E-03	4.99E-03	2.53E-03
	MAP1LC3B	1.56E-01	4.32E-31	1.78E-90	5.06E-70	8.43E-22	5.23E-33	3.88E-11	1.64E-47	9.35E-24
	GABARAPL1	3.14E-01	3.77E-14	1.07E-29	1.71E-61	7.49E-33	2.91E-35	1.79E-03	1.44E-06	6.22E-03
	ULK1	4.77E-01	1.62E-01	2.09E-04	5.66E-18	3.83E-16	1.94E-06	2.61E-02	1.18E-01	3.30E-03
	ATG4D	5.05E-01	8.60E-01	4.93E-01	1.55E-04	6.96E-07	8.39E-07	3.96E-02	1.39E-02	2.01E-01
	AMBRA1	2.31E-01	1.93E-02	8.13E-04	2.90E-11	2.42E-04	2.93E-02	9.29E-01	3.72E-01	5.88E-01
	SQSTM1	9.65E-01	6.80E-32	2.31E-82	0.00E+00	2.98E-299	1.17E-32	2.88E-233	2.15E-84	4.28E-159
	PIK3C3	1.62E-01	1.52E-03	7.32E-01	5.53E-05	1.25E-17	1.67E-04	2.63E-05	2.42E-07	1.19E-11
Lysosome	DRAM1	0.072547	0.1337506	6.92E-90	8.66E-94	1.1E-79	4.26E-41	1.8E-36	7.3E-28	9.2E-31
	FUCA1	9.46E-02	5.71E-01	7.90E-03	2.05E-04	2.92E-06	3.96E-09	1.82E-12	2.98E-11	3.75E-15
	DNASE2	3.68E-02	2.83E-01	6.65E-06	8.26E-08	5.78E-14	7.69E-12	7.71E-20	2.46E-22	2.05E-29
	ASAH1	8.97E-01	3.60E-01	1.06E-02	1.93E-02	1.71E-03	1.34E-03	2.28E-05	1.27E-08	4.36E-15
	LAMP3	6.41E-01	7.58E-01	9.85E-01	1.39E-01	5.44E-03	1.13E-02	1.70E-03	1.28E-09	4.12E-07
	PSAP	9.20E-01	6.34E-01	4.91E-22	3.85E-35	2.32E-38	4.15E-06	4.09E-31	3.15E-23	7.30E-63
	MANBA	6.94E-01	3.62E-07	1.89E-05	1.37E-01	2.18E-02	4.20E-01	1.21E-01	7.44E-02	1.04E-03
	IDS	5.16E-01	4.46E-01	8.26E-01	9.40E-01	4.49E-01	1.94E-01	7.46E-03	1.24E-01	6.68E-01
	MFSD8	7.02E-01	5.37E-01	4.62E-01	6.09E-01	2.62E-01	8.08E-02	2.55E-04	2.32E-03	3.35E-04
	ENTPD4	3.33E-01	7.38E-01	8.04E-02	5.37E-01	1.23E-02	1.54E-03	4.05E-04	3.27E-03	1.33E-03
	ABCA2	8.48E-01	7.74E-01	2.37E-04	9.00E-01	8.39E-01	6.89E-01	1.28E-01	3.32E-01	8.45E-05
	HEXA	4.25E-01	4.90E-01	4.13E-07	3.99E-06	2.86E-02	4.53E-03	6.62E-04	1.53E-04	5.18E-13
	CLN5	5.22E-01	3.85E-01	4.24E-01	5.40E-01	4.42E-01	7.85E-01	5.78E-03	2.28E-02	2.26E-10
	GALC	9.98E-01	2.86E-01	8.54E-01	5.04E-01	8.48E-01	2.89E-01	2.45E-02	2.77E-02	5.06E-06
	LAMP2	4.48E-01	2.56E-01	3.02E-05	4.71E-01	2.45E-02	5.31E-01	3.34E-06	1.23E-03	1.35E-11
	GALNS	9.51E-01	6.94E-01	4.74E-02	7.97E-01	8.06E-01	7.71E-01	5.73E-01	1.92E-01	2.44E-06
Antioxidant Response	NOS3	1.78E-01	1.47E-01	1.17E-31	6.28E-32	1.73E-05	1.89E-01	4.95E-01	1.01E-04	1.25E-03
	CAT	7.21E-01	8.66E-01	7.74E-04	8.61E-09	2.36E-06	7.48E-07	1.97E-02	6.97E-02	3.31E-01
	PRDX3	9.65E-01	2.60E-01	1.52E-01	6.76E-02	4.94E-08	1.49E-10	9.35E-15	7.05E-19	7.24E-12
	TXNRD2	7.01E-01	2.01E-04	1.48E-01	1.15E-01	1.30E-08	4.16E-02	1.11E-09	1.35E-03	9.89E-04
Stress Response TFs	KLF2	9.25E-11	2.80E-07	4.00E-06	9.28E-01	2.17E-01	2.81E-01	3.14E-05	6.45E-02	1.06E-09
	KLF4	8.41E-57	9.73E-49	1.58E-11	7.00E-05	7.21E-06	3.91E-08	9.72E-04	2.69E-12	4.46E-02
Thrombosis	THBD	8.54E-02	3.89E-11	1.57E-05	1.10E-01	3.40E-12	1.07E-22	2.56E-15	1.06E-25	3.58E-05

Supplementary Figure 4. Summary of $\log_2$ (fold-change) and p-values for genes included in Figure 2. Red indicates up-regulation, blue indicates down-regulation. The tiles' yellow coloring on the right indicates significant p-values ($p < 0{\cdot}05$). Pathways and processes corresponding to those mentioned in Figure 2 are listed in the first column.

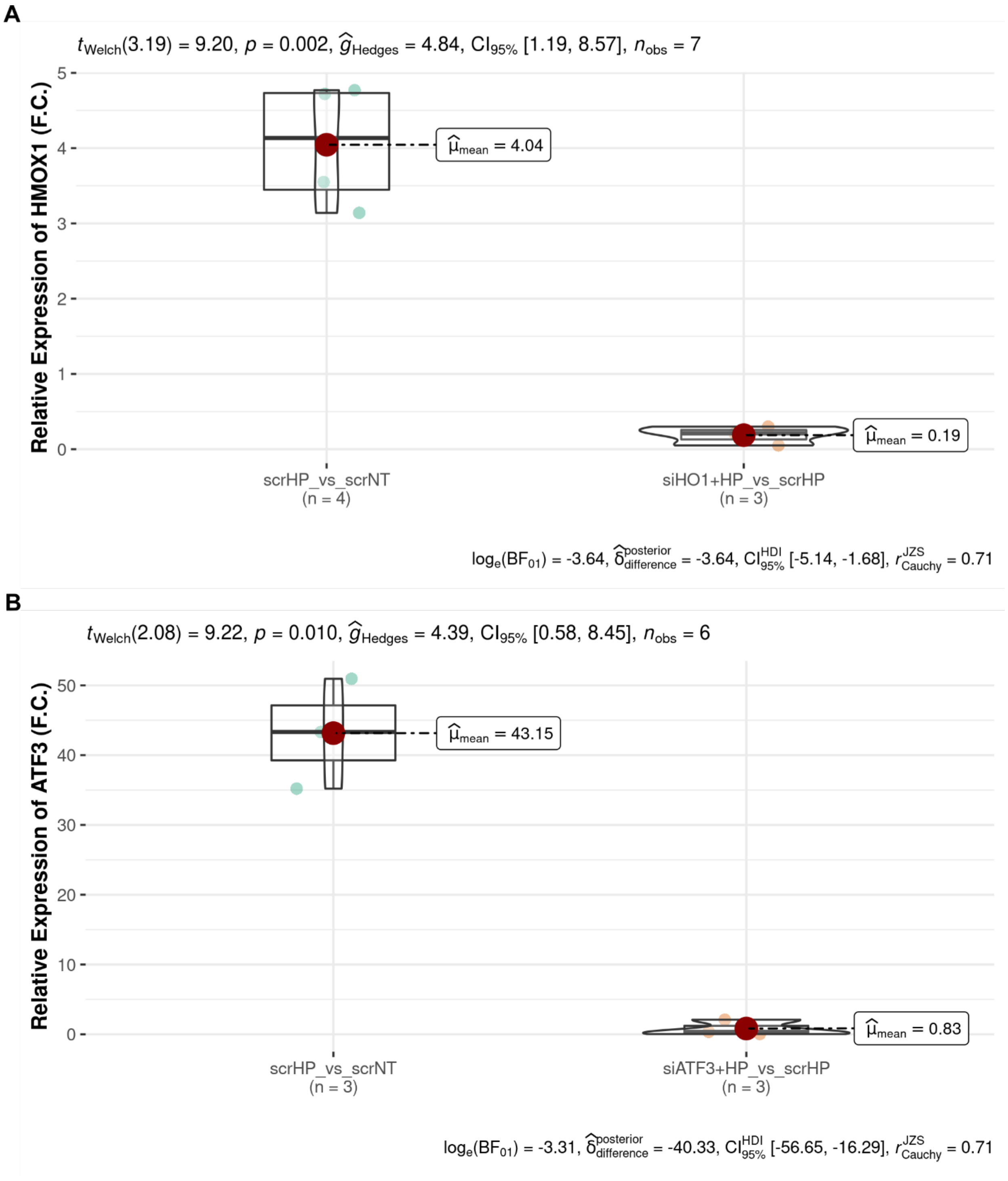

Supplementary Figure 5. Statistical analysis and plotting of HMOX1 and ATF3 knockdown under HP treatment. Frequentist analysis is shown on the top of the panel, and the $\log_e$ ratio of the Bayes factor, i.e., the ratio of the likelihood of null hypothesis (H_0) over the alternate hypothesis (H_1), is shown at the bottom. Both genes show a significant decrease in their expression upon their knockdown under HP treatment with a very strong effect size ($\hat{g} > 2$) and strong evidence for H_1 (BF >25).

Log2(Fold-Change)

		1h	2h	4h	6h	8h	10h	12h	14h	16h	scrHP vs. scrNT	siHMOX1+HP vs. scrHP
Cell Surface	AQP3	0.319952	0.691558	1.532701	1.435376	1.307273	1.284071	0.851665	0.404116	0.333059	1.21549	0.316385
	NOS3	0.126144	-0.16919	-0.80205	-1.02363	-0.744	-0.16525	0.067144	-0.32738	-0.26256	-0.62366	1.537879
	HMOX1	0.192624	2.336764	3.764534	4.052689	3.673319	2.78576	2.263246	2.937438	2.625416	3.092449	-2.97694
	CAV1	0.01451	0.036013	-0.11476	-0.34957	-0.47089	-0.51187	-0.08492	-0.37166	0.15684	-0.43438	-0.10461
	EGFR	-0.08961	-0.10747	0.297619	0.611133	0.343865	0.10752	0.106111	0.443331	0.014495	0.495901	-1.44578
	IGF1R	-0.00711	-0.21478	0.426468	1.415392	1.239511	1.083876	0.879769	0.786737	0.648099	0.810848	-0.92811
Mitochondria	atp5d	0.031249	-0.28411	-0.19539	-0.08111	-0.39746	-0.13629	-0.2592	-0.1212	-0.28898	-0.1295	0.664259
	atp5g1	-0.06122	-0.03949	-0.01758	-0.09568	-0.42102	-0.71538	-0.92429	-1.07132	-0.90462	-0.20805	0.461475
	atp5g3	0.005497	-0.00716	0.181993	0.045661	-0.14011	-0.25663	-0.22593	-0.24758	-0.05048	-0.1656	0.421624
	atp5i	-0.00594	0.007897	0.21045	0.230851	-0.04293	-0.15289	-0.2812	-0.31594	-0.20465	-0.1053	0.490012
	atp6ap1	0.046773	-0.10663	-0.04752	0.185101	0.040877	0.087192	0.089719	0.109991	0.197921	-0.09391	0.509871
	atp6v0a2	-0.04699	0.073951	-0.11689	-0.22529	0.056462	0.239497	0.142611	0.150122	-0.03971	0.237624	-0.42475
	atp6v0b	0.045647	0.094036	0.378906	0.326575	0.175944	0.409893	0.257524	0.223804	0.146646	0.275436	0.425804
	atp6v0c	0.131535	-0.07706	0.148271	0.530298	0.493212	0.588553	0.270899	0.324294	-0.0205	0.265325	0.459968
	atp6v0e1	0.011669	0.073135	0.169982	0.166924	0.147875	0.049562	0.171298	0.276358	0.230883	0.127802	0.515893
	atp6v0e2	0.023393	-0.08432	-0.19565	-0.3787	-0.6448	-0.53655	-0.53133	-0.55239	-0.57992	-0.19304	0.440653
	atp6v1b2	0.040342	0.235025	0.047748	0.364513	0.760077	0.720251	0.626218	0.808001	0.512361	0.746961	0.189166
	atp6v1c1	-0.04179	0.129127	0.029147	-0.10377	0.221474	0.189835	0.333975	0.395436	0.368473	0.225897	-0.59504
	atp6v1f	0.059568	-0.02742	0.156457	0.1435	-0.00749	0.128761	0.009014	0.053577	0.272309	0.10937	0.457655
	atp6v1g1	0.091019	0.218444	0.561232	0.773061	0.701193	0.559854	0.46054	0.564849	0.608958	0.441065	0.391363
	mt-atp6	-0.49367	-0.03245	-0.40821	-0.7159	-0.87636	-1.34516	-1.85374	-1.54796	-2.1445	-0.73521	0.409167
	mt-atp8	-0.77136	-0.26638	-0.57026	-1.07649	-0.93707	-1.63288	-2.05749	-1.70166	-2.36213	-1.03873	0.362516
	cox10	-0.19211	-0.35156	-0.48516	0.271826	0.530826	0.382821	0.14573	0.392177	0.013645	0.390345	-0.70164
	cox17	-0.02119	0.023988	0.373458	0.126141	-0.17268	-0.52951	-0.39078	-0.56239	-0.21834	-0.18345	0.67777
	cox5b	0.044964	-0.01882	0.250454	0.139436	-0.05495	-0.00833	-0.15509	0.001537	0.050498	-0.12428	-0.37625
	cox7a1	-0.19422	-0.13902	-0.23354	-0.11712	-0.29079	-0.36426	-0.51591	-0.24198	0.1268	0.052733	1.123867
	cox7a2	0.021294	0.055309	0.32304	0.354075	-0.01871	-0.08863	-0.236	-0.13787	-0.07434	0.137746	0.425521
	cox7a2l	0.024532	0.147679	0.666529	0.673647	0.534366	0.337096	0.382016	0.600425	0.470163	0.061265	0.553706
	cox8a	-0.01234	-0.04847	0.176401	0.249897	-0.09319	-0.09295	-0.33439	-0.33923	-0.54185	0.130272	0.679926
	mt-co1	-0.81722	-0.79247	-1.08867	-1.3768	-1.72326	-2.10884	-2.26194	-2.50303	-2.19081	-1.32899	0.010432
	mt-co2	-0.63383	-0.58248	-1.09097	-1.58682	-1.98871	-2.55859	-2.71686	-2.98944	-2.4804	-1.18836	0.28426
	mt-co3	-0.59943	-0.73339	-1.25541	-1.79706	-2.13125	-2.63641	-2.97425	-3.06712	-2.81313	-1.48037	0.233542
	mt-nd1	-0.45056	-0.22619	-0.6344	-0.5837	-0.8223	-1.0603	-1.07279	-1.28515	-0.9143	-0.75394	0.795651
	mt-nd2	-0.55543	-0.18693	-0.60377	-0.80395	-0.89956	-1.24949	-1.32813	-1.33602	-1.11147	-0.61502	0.66855
	mt-nd3	-0.63633	-0.73136	-1.338	-1.88085	-1.95809	-2.41633	-2.60105	-2.63694	-2.00915	-1.04949	0.7659
	mt-nd4	-0.51087	-0.21467	-0.5207	-0.749	-0.89598	-1.34514	-1.86559	-1.54245	-2.18148	-0.98303	0.592822
	mt-nd4l	-1.10659	-0.24635	-0.31448	-0.71129	-0.48832	-1.0411	-1.4673	-1.10537	-1.78367	-0.83248	0.60118
	mt-nd5	-2.03929	-0.76888	-0.70215	-0.78523	-0.89701	-1.31649	-1.80682	-1.40507	-1.92669	-0.96396	0.693583
	mt-nd6	-1.45398	-1.40066	-1.57602	-1.68949	-1.95137	-2.2324	-2.61022	-2.49336	-2.46448	-1.55829	0.341518
	ndufa1	0.087714	0.140297	0.448501	0.469945	0.325238	-0.0236	-0.02068	-0.00288	0.119482	0.112204	0.448612
	ndufa12	0.073153	0.029414	0.165951	0.269317	0.057294	-0.21515	-0.11562	0.080033	-0.15083	0.010512	0.635784
	ndufa4	0.095933	0.129794	0.362924	0.322948	0.121679	-0.11569	-0.03452	-0.0824	-0.01925	0.058773	0.385331
	ndufa4l2	0.589778	0.210943	2.03041	2.17777	1.570027	2.401499	1.555488	1.664355	2.119491	-0.15682	0.586698
	ndufa5	-0.10481	0.280518	0.205112	0.020758	-0.12044	-0.26377	-0.03202	-0.22228	0.205865	0.101295	0.446666
	ndufa7	-0.01148	-0.04919	-0.02767	0.071653	-0.29652	-0.33534	-0.53959	-0.4794	-0.3601	-0.25895	0.427506
	ndufab1	-0.03715	0.033711	0.152595	-0.01562	-0.25864	-0.46998	-0.43443	-0.31456	-0.34698	0.062612	0.577354
	nduf 1	0.109745	0.051326	0.353919	0.277825	-0.0683	-0.32065	-0.23108	-0.34765	-0.10085	-0.00082	0.458486
	nduf 10	0.042852	-0.0652	-0.24951	-0.0701	-0.20869	-0.23252	-0.24065	-0.04608	-0.32858	-0.12454	0.463041
	nduf 4	-0.06713	-0.0267	0.123318	0.125025	-0.14657	-0.23055	-0.33908	-0.25482	-0.35956	0.091161	0.371043
	nduf 5	0.050008	0.221541	0.449258	0.275849	0.247502	-0.13517	0.068852	0.027753	0.159266	0.016053	0.477603
	ndufv2	-0.02586	-0.02453	-0.05608	-0.09974	-0.17705	-0.36226	-0.24756	-0.21042	-0.31943	-0.1585	0.46281
	ppa1	-0.0383	0.069888	0.077763	-0.28392	-0.51004	-0.922	-0.64702	-0.63414	-0.42688	0.005969	0.419271
	ppa2	-0.02619	0.194954	0.195271	-0.13602	-0.28594	-0.61126	-0.45893	-0.55386	-0.1809	-0.00216	-2.54939
	sdhd	0.191273	0.17046	1.189252	0.249764	0.139659	NA	NA	NA	NA	NA	0.077721
	tcirg1	0.034435	-0.49859	-0.78633	-1.06213	-0.70265	-0.34565	-0.31714	-0.45244	0.049146	-0.2232	0.810006
	mt-cyb	-0.32714	-0.11452	-0.54691	-0.64575	-0.88592	-1.31724	-1.81996	-1.62982	-2.01583	-0.93072	0.539845
	uqcr10	-0.04529	-0.08379	-0.01219	-0.17025	-0.49906	-0.53296	-0.50064	-0.48097	-0.54765	-0.04882	0.329268
	uqcrc2	-0.02601	0.002256	-0.06258	-0.20602	-0.29296	-0.52268	-0.26656	-0.32755	-0.14612	0.095837	0.038401
	uqcr11	-0.01321	-0.05022	0.234121	0.111813	-0.00523	0.067522	-0.10039	-0.03424	0.048317	0.076666	0.350658
	uqcrfs1	-0.00469	-0.00163	0.148035	0.250534	0.103771	-0.15549	-0.15459	-0.07079	-0.19507	0.177832	0.272082
	uqcrb	0.02584	0.237043	0.432903	0.280345	0.208421	-0.21708	-0.07605	-0.11146	0.030994	0.161728	0.12094
	cyc1	0.047769	-0.05581	-0.03542	-0.06842	-0.30884	-0.3031	-0.35051	-0.28553	-0.18017	-0.02804	0.160104
	uqcrc1	-0.02408	-0.11438	-0.0677	-0.14192	-0.30643	-0.30627	-0.26199	-0.29454	-0.12571	-0.14048	-0.07917
	uqcrh	-0.02758	0.183815	0.368459	0.269298	0.072816	-0.00525	-0.0406	0.014272	0.030171	0.144727	0.082357
	uqcrq	-0.03654	-0.01382	0.272194	0.24471	-0.19819	-0.22197	-0.36819	-0.36925	-0.18631	0.013495	-0.29055
Nucleus	KDM6A	0.067121	-0.09674	1.589933	1.920636	1.581528	0.984873	0.647964	0.776189	0.497482	1.458672	-0.58335
	RBBP5	-0.1651	-0.20674	-0.26566	0.281707	0.360946	0.014556	0.065357	0.050941	0.07288	0.604658	-1.35231
	PPM1D	0.278276	0.949738	2.748199	2.859814	2.46043	1.747528	1.390247	1.282991	1.03867	2.573664	-0.24385
	LINC01747	-0.13423	-0.57053	-1.21406	-2.46101	-1.86904	0.077929	0.395117	0.323935	0.047092	-0.86456	1.025941
	LINC01091	-0.3534	-0.33216	-1.00287	-1.4603	-2.08766	-1.34884	-1.29149	-1.07241	-0.39345	-0.93905	1.335638
	SNHG15	0.300718	0.389207	1.422478	1.601392	1.439397	1.135658	0.430042	0.523827	-0.09407	1.446291	-0.71585
	PVT1	0.009888	0.030491	1.203739	1.731756	1.283189	1.136655	0.69593	0.863819	0.374815	1.034235	-0.57906
	EPB41L4A-AS1	-0.04015	0.388117	1.180677	1.424639	1.23483	0.889282	0.700365	0.821042	0.414509	0.966181	-0.40629
	CTC-499B15.5	-0.27841	0.026746	0.767378	1.069003	0.824293	-0.27479	-0.58017	-0.42905	-0.74589	0.801255	-0.50099
	NEAT1	0.040738	0.738442	1.43478	0.745106	0.931059	1.112758	0.629143	0.454094	-0.24376	0.52238	-0.39598
	RP11-54A9.1	-0.04541	0.043551	0.184645	0.451901	0.254945	0.096267	-0.04828	0.076322	-0.11934	0.282431	-0.3404

P-value

	1h	2h	4h	6h	8h	10h	12h	14h	16h	siHMOX1+HP vs. scrHP	scrHP vs. scrNT
AQP3	0.236374	0.006049	8.91E-11	2.41E-08	6.42E-08	2.48E-08	0.015907	0.153211	0.27185	2.46E-05	0.122676
NOS3	0.177763	0.147087	1.17E-31	6.28E-32	1.73E-05	0.188732	0.494992	0.000101	0.001247	1.66E-08	4.3E-116
HMOX1	0.090194	1.5E-154	0	0	9E-138	7.55E-44	9.3E-287	1.3E-203	0	0	0
CAV1	0.813271	0.563112	0.164287	5.11E-06	0.000029	3.88E-19	0.064022	6.96E-14	0.00541	1.45E-29	0.002221
EGFR	0.587811	0.496697	0.089103	0.000598	0.041924	0.503648	0.529641	0.062266	0.924543	0.003288	5.55E-18
IGF1R	0.950861	0.062349	0.017363	3.98E-20	1.32E-32	3.32E-18	2.39E-14	1.2E-12	8.85E-09	1.47E-19	3.09E-22
atp5d	0.732753	0.173274	0.052009	0.607234	0.0062	0.453991	0.024193	0.418576	0.013307	0.449505	2.81E-06
atp5g1	0.572198	0.799913	0.876944	0.464849	0.000767	1.9E-06	7.36E-14	2.96E-13	4.96E-14	0.072892	2.46E-09
atp5g3	0.942831	0.931828	0.033119	0.722559	0.107268	0.00628	0.02211	0.001345	0.562878	0.136591	1.19E-18
atp5i	0.949852	0.950452	0.02202	0.008091	0.625325	0.136741	0.000918	0.000105	0.005876	0.501638	6.03E-08
atp6ap1	0.324368	0.032742	0.578382	0.082686	0.392758	0.335359	0.109277	0.279578	1.48E-07	0.288347	9.94E-09
atp6v0a2	0.661669	0.501511	0.408707	0.11463	0.628553	0.027939	0.214755	0.280708	0.732047	0.02441	5.34E-06
atp6v0b	0.62769	0.304864	5.89E-05	0.011761	0.07711	0.000825	0.036934	0.02128	0.099699	3.38E-05	7.07E-16
atp6v0c	0.273687	0.714321	0.230114	0.000486	0.002607	0.000123	0.009514	0.012314	0.860919	0.079732	2.9E-06
atp6v0e1	0.913222	0.540773	0.11446	0.146874	0.216594	0.675601	0.147397	0.01074	0.028195	0.226505	1.47E-10
atp6v0e2	0.794295	0.289231	0.027226	0.000181	7.91E-11	0.0032	1.47E-05	5.31E-05	5.92E-10	0.016952	8.63E-12
atp6v1b2	0.545733	0.000773	0.470477	0.00066	1.68E-31	4.01E-23	3.68E-21	1.82E-35	9.98E-17	2.63E-39	9.89E-06
atp6v1c1	0.599808	0.058479	0.726535	0.418865	0.030872	0.181523	8.48E-06	0.000229	1.43E-08	9.57E-05	2.21E-31
atp6v1f	0.548505	0.709374	0.021781	0.07127	0.927596	0.389772	0.925277	0.506291	7.87E-06	0.224258	1.1E-07
atp6v1g1	0.207731	0.002205	4.46E-17	5.17E-22	1.83E-16	2.89E-11	2.04E-11	5.21E-14	2.23E-16	1.34E-17	1.4E-15
mt-atp6	9.13E-08	0.750165	0.008128	1.26E-05	4.96E-13	9.67E-31	4.18E-49	4.45E-59	4.48E-68	4E-30	2.94E-08
mt-atp8	2.79E-13	0.023204	0.004137	4.31E-15	3.31E-06	7.31E-36	2.73E-48	6.28E-42	6.7E-79	9.66E-61	2.22E-08
cox10	0.150426	0.005284	0.008475	0.075773	1.94E-05	0.001777	0.269372	0.004818	0.938163	0.011847	5.46E-07
cox17	0.864868	0.870735	0.00185	0.349643	0.222492	0.000419	0.007016	0.000163	0.077171	0.159043	6.53E-12
cox5b	0.65545	0.813704	0.001965	0.126069	0.525455	0.953584	0.148199	0.989408	0.518265	0.326354	0.00116
cox7a1	0.321955	0.432688	0.242866	0.593618	0.192944	0.09565	0.02549	0.27104	0.47024	0.717145	1.26E-27
cox7a2	0.779685	0.481969	9.86E-05	0.003508	0.844038	0.324523	0.021686	0.089793	0.36312	0.258305	7.7E-08
cox7a2l	0.770535	0.048166	2.59E-19	6.23E-12	6.6E-06	8.68E-05	7.64E-06	2.95E-13	2.97E-11	0.349703	7.82E-36
cox8a	0.880241	0.544344	0.011684	0.001787	0.296867	0.541961	8.68E-05	0.001011	1.15E-14	0.025376	8.9E-47
mt-co1	2.16E-13	7.34E-12	5.79E-12	1.33E-14	2.55E-44	1.89E-55	2.59E-95	9.8E-127	2.55E-60	5.84E-60	0.884871
mt-co2	7.55E-08	5.45E-06	8.08E-12	1.96E-26	1.64E-35	6.53E-74	1E-111	4.3E-141	1.3E-87	4.2E-43	7.47E-06
mt-co3	5.1E-07	6.21E-12	2.02E-21	2.03E-53	4.25E-44	2.05E-73	2.4E-179	1.4E-189	4E-118	2.19E-94	0.002032
mt-nd1	0.018868	0.36693	0.014436	0.064981	0.000259	7.44E-06	5.88E-07	4.24E-10	4.33E-05	2.78E-58	1.1E-112
mt-nd2	9.06E-12	0.030876	0.000127	1.07E-08	3.3E-11	2.57E-29	2.52E-54	1.13E-55	2.83E-36	9.82E-38	6E-29
mt-nd3	1.13E-08	9.52E-17	6.07E-39	5.82E-87	1.69E-30	2.67E-65	2.01E-79	6.4E-142	2E-120	5.05E-17	4.1E-22
mt-nd4	6.64E-05	0.156891	0.002803	0.000351	5.9E-11	4.14E-21	2.61E-30	4.33E-36	1.47E-55	2.8E-212	2.87E-24
mt-nd4l	1.71E-22	0.00343	0.029801	2.93E-14	3.54E-05	6.83E-28	4.81E-37	1.57E-36	1.22E-53	1.38E-19	3.06E-16
mt-nd5	1.16E-83	2.5E-13	5.66E-06	0.000126	2.51E-16	1.76E-39	8.63E-46	4.04E-55	7.67E-74	6.4E-147	4.15E-62
mt-nd6	3.84E-11	3.28E-17	1.37E-15	2.59E-15	7.51E-33	3.51E-40	7.17E-53	3.64E-64	4.75E-59	2.29E-66	7.01E-12
ndufa1	0.241324	0.109046	8.73E-05	9.24E-10	8.54E-05	0.775121	0.780837	0.970209	0.124064	0.398465	1.98E-12
ndufa12	0.436864	0.758843	0.080645	0.042272	0.560922	0.083975	0.289595	0.439879	0.099509	0.931786	4.79E-20
ndufa4	0.166073	0.082721	1.11E-08	3.94E-06	0.073276	0.199398	0.664376	0.20175	0.72169	0.533653	1.67E-17
ndufa4l2	0.032808	0.372355	0.001124	0.000183	0.002949	1.83E-06	0.000621	0.000725	1.44E-06	0.717372	0.12908
ndufa5	0.243348	0.048972	0.109022	0.860238	0.485527	0.134153	0.743146	0.010435	0.00242	0.210004	4.2E-09
ndufa7	0.938392	0.743719	0.834366	0.610122	0.042784	0.030575	0.000595	0.016268	0.00846	0.004581	1.36E-07
ndufab1	0.763018	0.791638	0.2202	0.910564	0.052842	0.003668	0.005302	0.013449	0.008422	0.441263	3.9E-20
nduf 1	0.396827	0.64625	0.009155	0.050154	0.44264	0.01435	0.052082	0.005847	0.472019	0.99585	4.69E-07
nduf 10	0.571673	0.358539	0.030155	0.617531	0.010306	0.009063	0.001147	0.547093	5.14E-07	0.319295	0.000192
nduf 4	0.392831	0.66582	0.126515	0.167991	0.019015	0.000349	0.001468	6.89E-05	4.6E-07	0.125561	6.61E-14
nduf 5	0.58016	0.063082	0.000257	0.011812	0.051042	0.230854	0.491769	0.784005	0.054396	0.887043	1.18E-20
ndufv2	0.770232	0.699806	0.441478	0.441837	0.011573	0.001626	0.003196	0.038474	1.23E-06	0.017151	6.2E-25
ppa1	0.469925	0.147325	0.120562	6.48E-07	2.96E-06	2.95E-23	8E-28	3.48E-26	3.12E-17	0.938453	7.5E-25
ppa2	0.848421	0.176552	0.150071	0.360295	0.050048	0.000395	0.005134	0.000414	0.186681	0.983636	2.8E-128
sdhd	0.265501	0.432891	0.043144	0.432706	0.540175	NA	NA	NA	NA	NA	0.574502
tcirg1	0.789865	0.0004	9.5E-08	2.39E-10	0.001519	0.014381	0.02786	0.001925	0.766559	0.347982	2.26E-06
mt-cyb	0.253507	0.621934	0.026285	0.028241	5.73E-05	8.42E-09	4.47E-16	3.89E-16	1.67E-20	6.2E-99	1.82E-18
uqcr10	0.654484	0.346984	0.917393	0.240334	1.9E-06	1.44E-06	2.33E-06	2.44E-06	2.3E-09	0.675472	5.99E-06
uqcrc2	0.614679	0.95937	0.306186	0.014887	3.83E-05	1.62E-09	2.91E-07	2.34E-09	0.001064	0.113892	0.393235
uqcr11	0.915968	0.687782	0.063053	0.429735	0.966831	0.69938	0.487132	0.789722	0.692499	0.332747	4.65E-06
uqcrfs1	0.960192	0.988551	0.114118	0.013363	0.325768	0.175626	0.274988	0.48256	0.036264	0.002746	2.48E-09
uqcrb	0.77348	0.05911	0.000217	0.004784	0.067796	0.235257	0.469708	0.250254	0.733736	0.222325	0.086717
cyc1	0.40053	0.330075	0.538892	0.510443	1.93E-07	0.008439	0.000403	0.006121	0.023405	0.66497	0.002995
uqcrc1	0.758562	0.024522	0.208797	0.011442	9.42E-09	0.017437	0.017621	0.001377	0.003479	0.01528	0.105397
uqcrh	0.748835	0.026869	1.89E-05	0.004403	0.506091	0.955885	0.676458	0.869114	0.728774	0.179877	0.034162
uqcrq	0.664155	0.867244	0.006065	0.008029	0.039716	0.018692	0.003382	0.000104	0.047803	0.883425	2.64E-08
KDM6A	0.639673	0.479732	4.83E-37	3.12E-38	2.79E-16	6.45E-12	3.05E-05	4.16E-06	0.000225	1.18E-25	1.67E-09
RBBP5	0.10336	0.025525	0.009303	0.013954	0.001199	0.883929	0.503482	0.617653	0.399044	0.000277	1.35E-16
PPM1D	0.022325	7.67E-17	9.9E-184	9.8E-118	2.87E-51	1.5E-42	9.79E-37	1.5E-29	8.67E-19	1.2E-100	0.00152
LINC01747	0.702908	0.163765	0.037023	2.22E-05	0.000473	0.844778	0.307871	0.484923	0.900205	0.046574	0.015686
LINC01091	0.224578	0.289987	0.012324	0.000482	6.59E-06	0.001463	0.000467	0.012322	0.371085	0.027116	0.000537
SNHG15	0.052879	0.000337	2.41E-35	7.11E-51	6.31E-41	1.34E-11	0.000831	1.44E-05	0.506579	2.06E-62	1.71E-29
PVT1	0.937914	0.794508	1.43E-27	1.75E-21	2.58E-19	5.48E-22	1.02E-08	4.59E-13	0.015261	1.25E-21	2.23E-11
EPB41L4A-AS1	0.792773	0.026403	1.56E-12	6.98E-23	2.86E-22	1.84E-13	7.33E-08	1.46E-09	0.000476	6.13E-23	1.82E-05
CTC-499B15.5	0.398832	0.941439	0.044331	0.004556	0.019383	0.469904	0.149991	0.295018	0.047384	0.004882	0.041081
NEAT1	0.90737	1.31E-06	1.11E-18	0.000233	0.000475	9.78E-05	0.171931	0.109917	0.420713	2.59E-39	1.25E-07
RP11-54A9.1	0.614632	0.666902	0.141208	0.001823	0.009606	0.30219	0.601022	0.482096	0.311175	0.002754	1.65E-05

Supplementary Figure 6. Summary of log2 (fold-change) and p-values for genes included in Figure 5. Red indicates up-regulation, blue indicates down-regulation. The tiles' yellow coloring on the right indicates significant p-values ($p<0{\cdot}05$). Cellular locations mentioned in FIG 5 are listed in the first column.

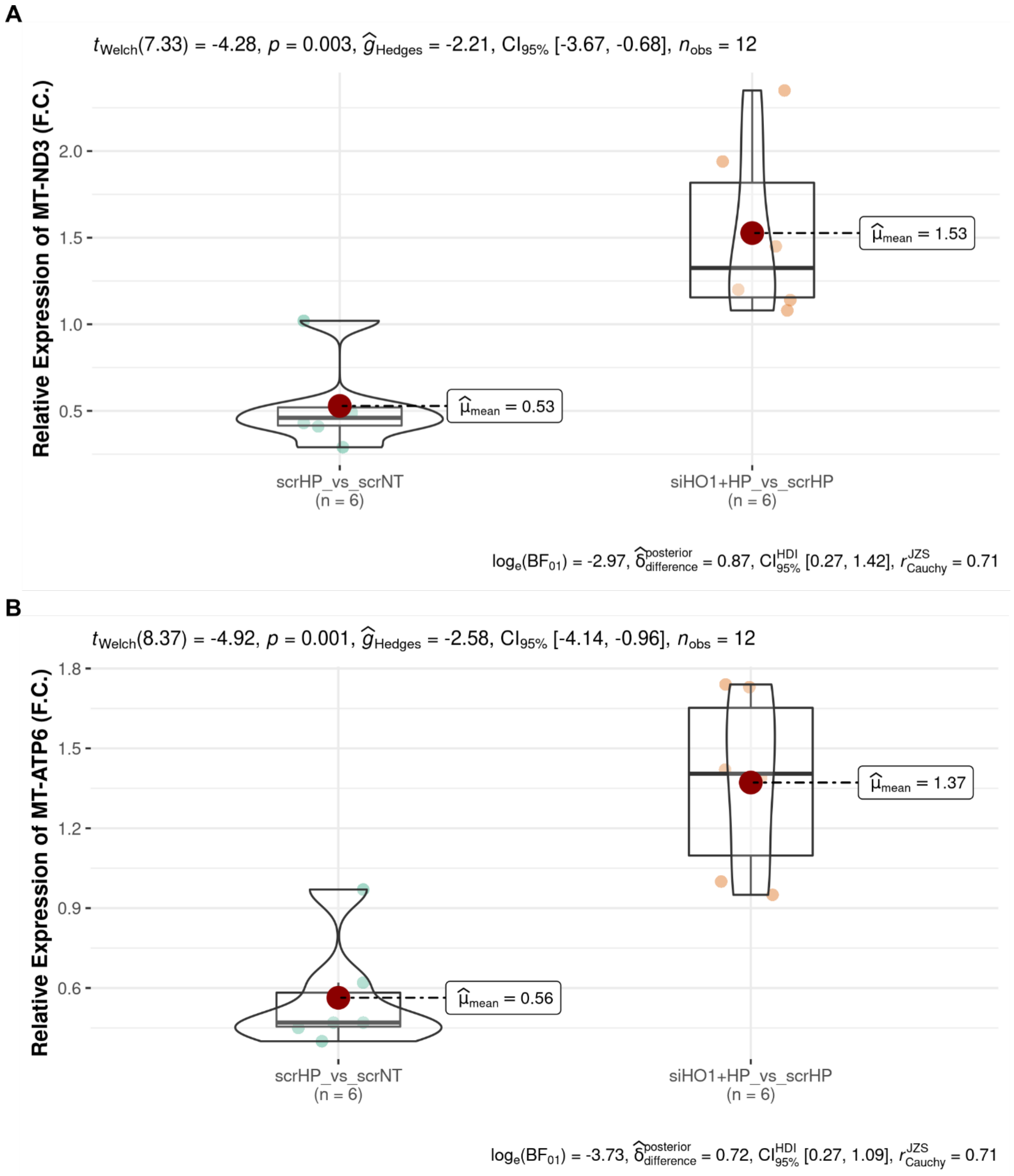

Supplementary Figure 7. Statistical analysis and graphing of mitochondrial transcripts following HMOX1 KD. Both mitochondrial ETC genes are significantly upregulated ($p < 0.003$) following HMOX1 KD upon HP treatment with a strong effect size ($\hat{g} > 2$) and a strong likelihood for the H_1 ($BF_{01} > 10$).

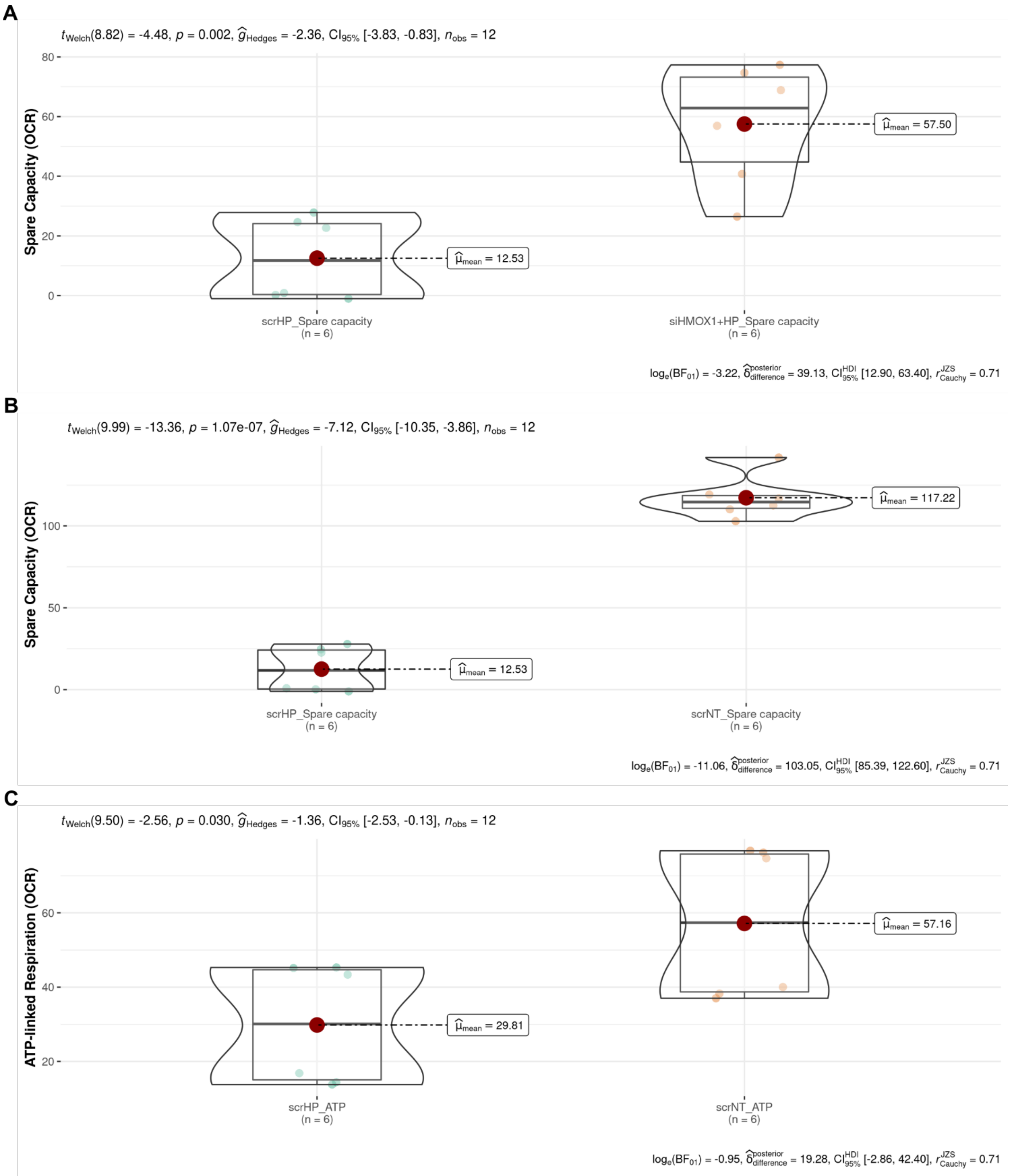

Supplementary Figure S8. Mitochondrial respiratory capacity measurements. A-B: The spare capacity (respiratory reserve) is significantly different among scrNT vs. scrHP and scrHP vs. siHOX1+HP groups with very strong effect size ($\hat{g}$>2). **C.** There is a significant decrease (p =0.03) in ATP production upon HP treatment in scrambled group, with modeate effect size.

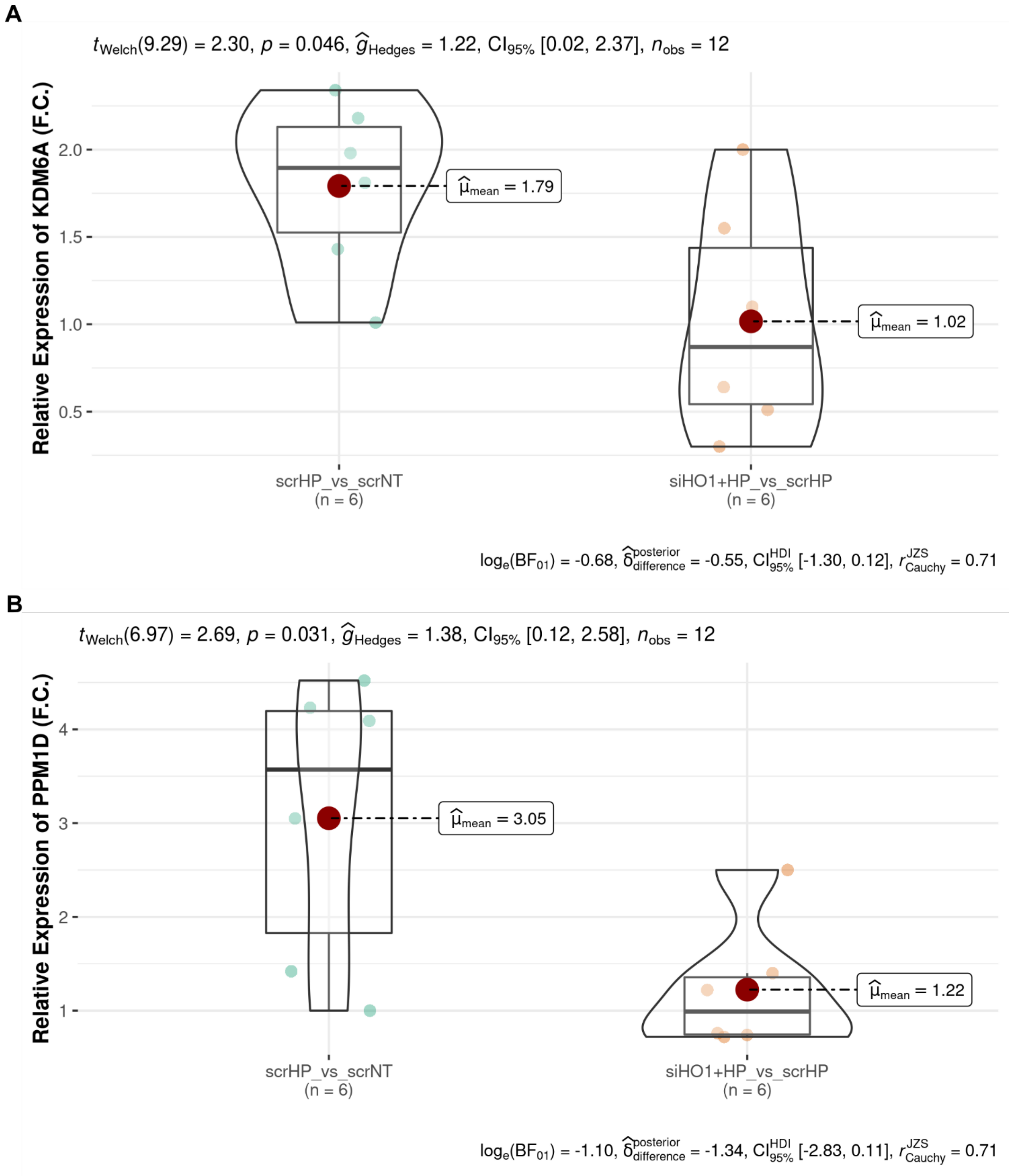

Supplementary Figure 9. Statistic analysis of epigenetic modifiers following HMOX1 KD under hydrogen peroxide treatment. Both epigenetic modifiers have switched their direction of expression with significant p-value with moderate effect size and mild evidence for H_1**.**

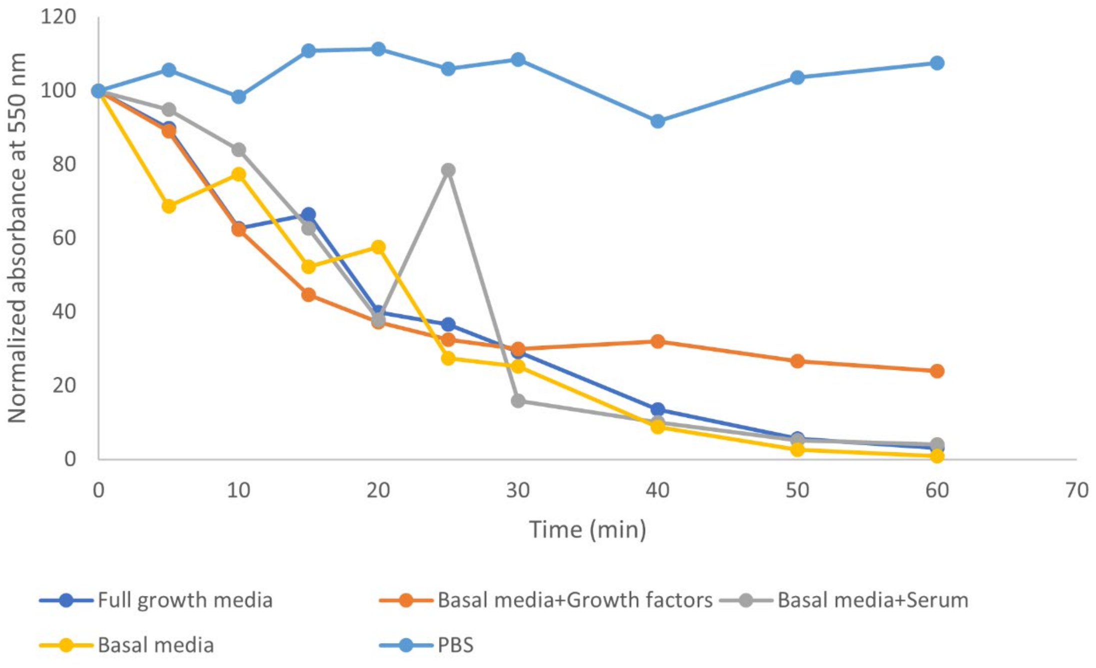

Supplementary Figure 10. Temporal stability of hydrogen peroxide in different media compositions. The biochemical stability of 100 µM HP was plotted as a normalized absorbance with time. The absorbance values indicate the amount of HP in the media. In full growth media (light blue line), HP is reduced drastically with time (<50% in 20 min) and disappears in about 60 min. A similar trend was observed for other media solutions except for PBS, where HP is stable over the tested period. In general, the rate of HP clearance depends on cell type, passage number, culture conditions like cell density, media volume, media type, etc.[92] In the case of primary HUVECs, the rate of HP clearance depends on several factors, including race, gender, age, and clinical profile (smoking status, environmental exposures, underlying health) of the EC donors in addition to the culture conditions. We believe a single bolus of 0·5 mM HP induction perturbs the cell and places it at the junction of cell survival and death and serves as a model to study both early and late phase OSR.
